# A DEAD-box helicase drives the partitioning of a pro-differentiation NAB protein into nuclear foci

**DOI:** 10.1038/s41467-023-42345-9

**Published:** 2023-10-18

**Authors:** Akiko Doi, Gianmarco D. Suarez, Rita Droste, H. Robert Horvitz

**Affiliations:** Howard Hughes Medical Institute, Department of Biology, Massachusetts Institute of Technology, Cambridge, MA 02139 USA

**Keywords:** Gene regulation, Development, Stem-cell differentiation

## Abstract

How cells regulate gene expression in a precise spatiotemporal manner during organismal development is a fundamental question in biology. Although the role of transcriptional condensates in gene regulation has been established, little is known about the function and regulation of these molecular assemblies in the context of animal development and physiology. Here we show that the evolutionarily conserved DEAD-box helicase DDX-23 controls cell fate in *Caenorhabditis elegans* by binding to and facilitating the condensation of MAB-10, the *C. elegans* homolog of mammalian NGFI-A-binding (NAB) protein. MAB-10 is a transcriptional cofactor that functions with the early growth response (EGR) protein LIN-29 to regulate the transcription of genes required for exiting the cell cycle, terminal differentiation, and the larval-to-adult transition. We suggest that DEAD-box helicase proteins function more generally during animal development to control the condensation of NAB proteins important in cell identity and that this mechanism is evolutionarily conserved. In mammals, such a mechanism might underlie terminal cell differentiation and when dysregulated might promote cancerous growth.

## Abstract (web summary)

The mechanism of spatiotemporal gene regulation during animal development is a fundamental question in biology. Here the authors show that the DEAD-box helicase DDX-23 controls stem cell fate by driving the formation of NAB repressive transcriptional condensates.

## Introduction

During animal development, gene regulation must be temporally precise for proper cell-fate decisions to occur. The evolutionarily conserved heterochronic gene pathway regulates multiple aspects of developmental timing and governs multiple cell-fate decisions. Heterochronic genes were first discovered in *C. elegans* from genetic screens for mutants with temporal transformations in the cell fates of a stem cell-like population of hypodermal seam cells^1^. The *C. elegans* heterochronic genes execute a temporally regulated larval developmental program that drives a precise succession of events, culminating in the terminal differentiation of the seam cells and the onset of adulthood^2–5^. The mammalian homologs of these genes play critical roles in various aspects of physiology and development, including metabolism, stem-cell regulation, and fertility^6–10^. The dysregulation of heterochronic genes has been implicated in multiple cancers^6–13^, including chronic myeloid leukemia (CML)^12^ and hepatocellular carcinoma (HCC)^14^.

**Fig. 1:**
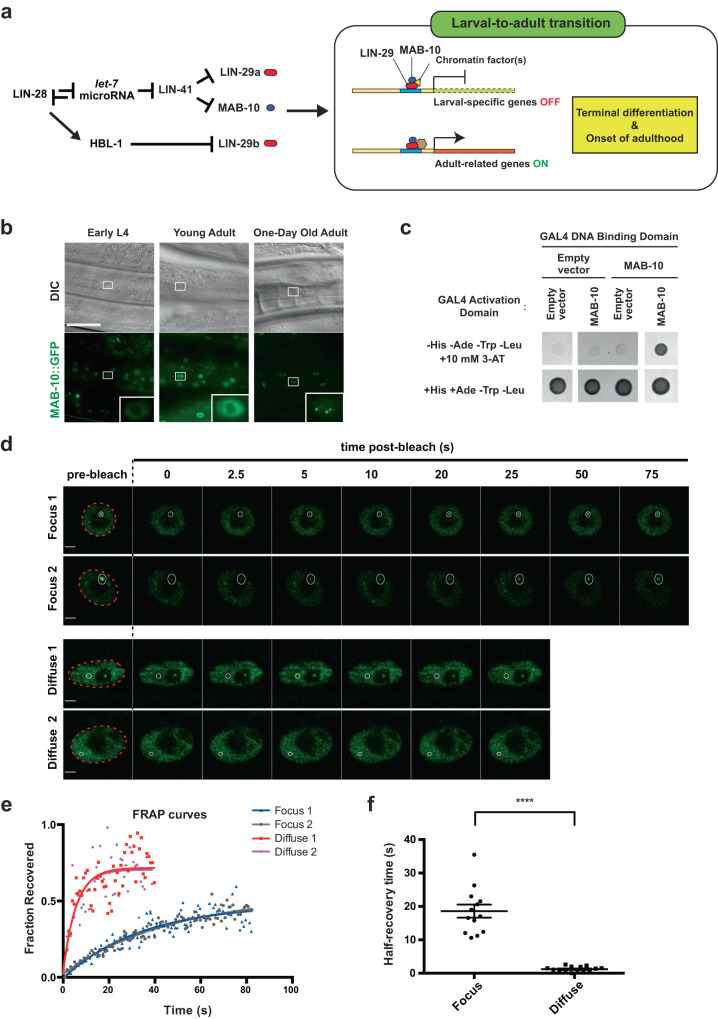
MAB-10 protein forms dynamic nuclear foci. **a** Known gene regulatory interactions in the heterochronic pathway. LIN-29 (red) and MAB-10 (blue) act together on a subset of LIN-29 target genes to downregulate larval-specific genes and turn on adult-related genes. **b** Differential interference contrast (DIC) and confocal GFP fluorescence images of hypodermal cells in early L4, young adult, and 1-day-old adult wild-type animals expressing the translational reporter *n5909 [mab-10::gfp]*, which tags the endogenous *mab-10* gene locus with *gfp*. Images are representative of ten animals. Scale bar, 40 μm. **c** Yeast two-hybrid spot assay showing the self-interaction of MAB-10 protein (upper right spot) in quadruple drop-out plates (-His/-Ade/-Trp/-Leu) containing the competitive inhibitor of histidine synthesis 3-AT. **d** Representative confocal fluorescent images of individual frames from in vivo fluorescence recovery after photobleaching (FRAP) time-lapse studies of two MAB-10 nuclear foci (“Focus”) and two diffuse MAB-10::GFP signals (“Diffuse”) in hypodermal nuclei of 1-day-old wild-type adults expressing the reporter *n5909 [mab-10::gfp]*. Images are representative of *n* = 13 (for “Focus”) and *n* = 14 (for “Diffuse”) independent experiments examining the MAB-10::GFP signal. Dotted red circle, nuclear circumference; white circle, focal area of laser bleaching and region of measurement; scale bars, 2 μm. **e** FRAP curves quantifying observations from the experiments shown in (**c**) (two MAB-10 nuclear foci (“Focus”, red and pink) and two diffuse MAB-10::GFP signals (“Diffuse”, blue and gray) in hypodermal nuclei). Fluorescence intensity measurements in the area of laser bleaching, normalized by considering the pre-bleach fluorescence intensity to be 1 and post-bleach intensity to be 0, are shown. *t* = 0, time measuring pre-bleaching intensity. **f** Half-maximal recovery times from FRAP data for 13 experiments examining MAB-10::GFP in foci (“Focus”) and 14 experiments examining MAB-10::GFP diffuse signal (“Diffuse”). Error bars, mean value +/− SEM. ****P = 1.278 × 10^−6^ (two-sided *t* test). Source data for (**e**, **f**) are provided as a Source Data file.

During *C. elegans* development, the heterochronic gene *lin-28* is expressed during the early larval stages and its protein product, LIN-28, inhibits the processing of the *let-7* microRNA (Fig. 1a)^1,4,13,15^. LIN-28 levels drop, and in the fourth and final larval stage, *let-7* microRNA levels are upregulated, silencing LIN-41 and thereby relieving *lin-29a* repression by LIN-41^16–18^. LIN-28 also acts independently of *let-*7 to downregulate *lin-29b* levels via the *hbl-1* gene during the first three larval stages^18^. During the fourth larval stage, these coordinated LIN-28 activities no longer inhibit LIN-29, which then drives exit from the (stem) cell cycle, terminal differentiation and the larval-to-adult transition^16,19^. LIN-29 is an early growth response (EGR) protein that acts in part with the NGFI-A-binding protein (NAB) transcriptional co-factor MAB-10 both to promote the expression of genes that drive terminal hypodermal seam cell differentiation and repress the expression of larval-specific hypodermal genes and to drive the onset of adulthood^20^. This process is evolutionarily conserved. For example, in mammals EGR proteins interact with NAB proteins to regulate terminal differentiation in multiple lineages^21,22^. EGR proteins have also been implicated in the regulation of luteinizing hormone LHβ expression in the pituitary gland, a key hormone involved in the onset of puberty^8,23^. Furthermore, EGR1, the mammalian homolog of LIN-29, is a barrier to the reprogramming of somatic cells into human induced pluripotent stem cells (iPSCs)^7^, highlighting the key involvement of this gene in controlling stem-cell fates. Despite the importance of the heterochronic pathway, mechanisms that regulate the transcriptional activities of the terminal effectors LIN-29 (EGR) and MAB-10 (NAB) remain largely unknown.

To better understand mechanisms that control the activity of the *C. elegans* developmental timing NAB protein MAB-10, we analyzed MAB-10 genetically and biochemically. Here we report that the formation of MAB-10 nuclear foci is facilitated by the DEAD-box helicase protein DDX-23, which binds to the MAB-10 protein and functions as a regulator of stem-cell fate. We propose that MAB-10 protein condensates control the transcriptional landscape in terminally differentiated seam cells. We further propose that the interaction between DDX-23 and MAB-10 is evolutionarily conserved, that a similar mechanism regulates mammalian stem-cell development and the onset of puberty, and that misregulation of this process can promote cancer progression.

## Results

### MAB-10 protein forms nuclear foci in vivo

To better define the role of MAB-10 during *C. elegans* development, we characterized the MAB-10 expression pattern and MAB-10 protein dynamics. To examine MAB-10 expression and localization under native regulatory control, we endogenously tagged the C-terminus of the *mab-10* genomic locus with GFP using the CRISPR-Cas9 genome editing technology. The MAB-10::GFP animals did not display any of the phenotypic abnormalities characteristic of *mab-10(lf)* mutants, indicating that this GFP-tagged *mab-10* locus maintains *mab-10* function. MAB-10::GFP expression turned on in the nuclei of two classes of epithelial-like cells—hypodermal cells and seam cells—in the early L4-stage. As the animals completed the larval-to-adult transition and became young adults, MAB-10::GFP expression increased and became localized throughout nuclei as well as in nuclear foci. In 1-day-old adults, most of the MAB-10::GFP signal was localized to nuclear foci (Fig. 1b). The tissue expression patterns as well as the subcellular localization of MAB-10::GFP were in agreement with previous work from our laboratory studying a transgenic strain expressing MAB-10::GFP in a *mab-10(lf)* background^20^.

Protein self-interaction is a common feature of proteins found in nuclear bodies^24,25^. Using a yeast two-hybrid spot assay, we showed that MAB-10 proteins can self-interact (Fig. 1c), suggesting that MAB-10 self-association might facilitate MAB-10 localization to nuclear foci. To examine the dynamic properties of the MAB-10 nuclear foci, we performed fluorescence recovery after photobleaching (FRAP) studies and time-lapse imaging of MAB-10::GFP foci in vivo using 1-day-old adults (Fig. 1d). We found that MAB-10::GFP foci recovered fluorescence more slowly after photobleaching than did diffuse MAB-10::GFP in the hypodermal nuclei in which the fluorescence was distributed throughout the nucleus (Fig. 1d, e). The half-maximal fluorescence recovery time, a measure of the MAB-10 protein exchange rate between the photobleached spot and the surrounding environment, was substantially longer for foci (*t*_1/2_ of ~18.6 s) than for the diffuse MAB-10::GFP signal in the hypodermal nuclei (*t*_1/2_ of ~1.2 s) (Fig. 1f). The reduced mobility of MAB-10 proteins in nuclear foci as revealed by FRAP data suggests that diffusion barriers affect kinetic properties of the molecules within these structures.

### DDX-23 is a regulator of stem-cell fate in *C. elegans* hypodermal seam cells

**Fig. 2:**
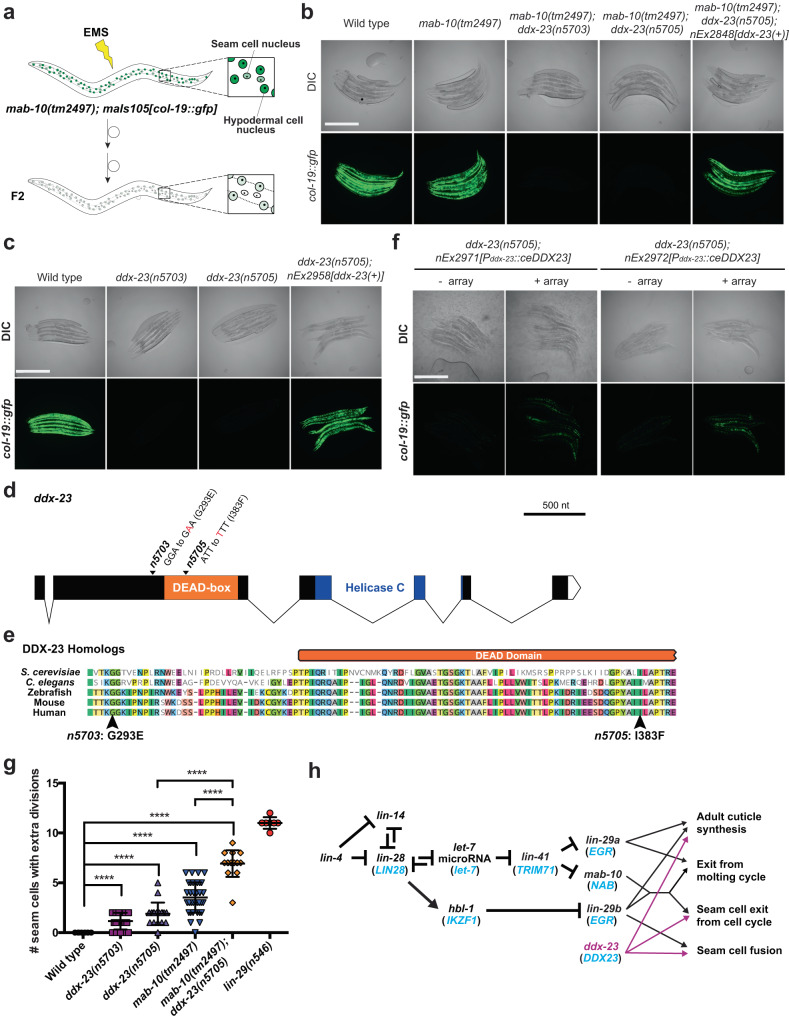
DDX-23 is evolutionarily conserved and functions in hypodermal stem cell biology. **a** Schematic of the F2 non-clonal *mab-10* enhancer screen to identify factors that act with or in parallel to MAB-10. *mab-10(tm2497)* mutants were ethyl methanesulfonate (EMS)-mutagenized, and F2 progeny were screened for second-site mutations that further reduced the low hypodermal *col-19* expression levels of *mab-10(tm2497)* mutants. **b** The *mab-10* enhancer screen isolated two alleles (*n5703* and *n5705*) of *ddx-23*. The *ddx-23(+)* transgene *nEx2848*, which contains wild-type *ddx-23*, rescued the low *maIs105* GFP expression. Representative DIC and GFP fluorescence micrographs of five animals per genotype are shown of *C. elegans* adults of the genotypes indicated and grown at 20 °C. Scale bar, 500 μm. **c** Representative DIC and GFP fluorescence micrographs of wild-type and *ddx-23* single mutant adult animals grown at 20 °C. Images are representative of five animals per genotype. The *ddx-23(+)* transgene *nEx2958*, which contains wild-type *ddx-23* tagged with *tagRFP-T*, rescued the low *maIs105* GFP expression. Scale bar, 500 μm. **d** Schematic of *ddx-23* gene structure. DEAD-box domain (orange) and helicase C domain (blue) are depicted. The *ddx-23(n5703)* and *ddx-23(n5705)* mutations isolated from the *mab-10* enhancer screen are indicated. **e** Sequence alignments of DDX-23 homologs from *S. cerevisiae* (Prp28), zebrafish (Ddx23), *Mus musculus* (DDX23), and *Homo sapiens* (DDX23). Only the region surrounding amino acid residues G293 and I383, which are altered by the *ddx-23* mutations *n5703* and *n5705*, respectively, is shown. Arrows indicate the completely conserved G293 and I383 residues. **f** Representative micrographs of *maIs105[col-19::gfp]* reporter GFP expression in 1-day-old *ddx-23(n5705)* adults with or without the transgene overexpressing human *DDX23* (*ceDDX23*). Images are representative of 5–8 animals per genotype. Scale bar, 500 μm. **g** Number of seam cells that underwent extra seam cell division, scored in 1-day-old adults in wild-type (*n* = 8), *ddx-23(n5703)* (*n* = 19), *ddx-23(n5705)* (*n* = 18), *mab-10(tm2497)* (*n* = 36), *mab-10(tm2497);ddx-23(n5705)* (*n* = 15), and *lin-29(n546)* (*n* = 7) animals. Error bars, mean value +/− SD. ****P < 0.0001 (two-sided *t* test). *P* = 1.0 × 10^−5^ (wild-type vs. *ddx-23(n5703)*), *P* = 1.8 × 10^−6^ (wild-type vs. *ddx-23(n5705)*), *P* = 7.8 × 10^−16^ (wild-type vs. *mab-10(tm2497)*), *P* = 9.9 × 10^−12^ (wild-type vs. *mab-10(tm2497);ddx-23(n5705)*), *P* = 8.8 × 10^−9^ (*mab-10(tm2497)* vs. *mab-10(tm2497);ddx-23(n5705)*), and *P* = 5.6 × 10^−12^ (*ddx-23(n5705)* vs. *mab-10(tm2497);ddx-23(n5705)*). **h** Proposed role of the *ddx-23* gene in the heterochronic pathway. DDX-23 acts with MAB-10 to control seam cell exit from the cell cycle but acts independently of MAB-10 to control seam cell fusion in adults. The human homologs of the *C. elegans* heterochronic genes are indicated in blue. Source data for (**g**) is provided as a Source Data file.

While *lin-29* mutants fail at all of the events that characterize the larval-to-adult transition, *mab-10* mutants fail at only a subset of these events^20^ (Supplementary Fig. 1a), suggesting that there are factors that act with or in parallel to MAB-10 in controlling terminal seam cell differentiation and the onset of adulthood. To identify such factors, we performed a mutagenesis screen using a reporter for the adult-specific *col-19* collagen gene, a downstream target gene of the heterochronic pathway that is highly expressed in the adult hypodermis^3,26^. In wild-type adults the *col-19::GFP* fluorescent reporter *maIs105* is upregulated, whereas in *lin-29* mutant adults this reporter is not expressed. In *mab-10* mutant adults *col-19::GFP* expression is reduced relative to that in wild-type animals (Supplementary Fig. 1b). We screened *mab-10(lf)* mutants for second-site mutations that further reduced their low *col-19* expression level (Fig. 2a). From this screen, we isolated two causal mutations (*n5703* and *n5705*) that proved to be distinct alleles of the gene *ddx-23* (Fig. 2b and Supplementary Fig. 1c). Each of these *ddx-23* mutations alone caused reduction of *col-19*::GFP expression in adult hypodermal cells in a wild-type background (Fig. 2c and Supplementary Fig. 1d). Expressing a wild-type copy of the *ddx-23* gene (using *ddx-23(+)* transgenes) in worms with *ddx-23* mutations restored *col-19* expression (Fig. 2b, c). We note that the strain with the mutation *ddx-23(n5705)* carried two different DDX-23 missense mutations, S365N and I383F. We recreated these two mutations individually using CRISPR-Cas9, examined the phenotypes of the two single-mutant strains (Supplementary Fig. 2a–d), and concluded that the DDX-23[I383F] mutation was responsible for the visible phenotypic abnormalities of the original screen isolate (Fig. 2c, g and Supplementary Fig. 2b–d). For simplicity, we refer to this mutation as *n5705*. These observations established that *ddx-23* loss-of-function reduces *col-19* expression levels, and that *ddx-23*, like *lin-29*, is required for *col-19* upregulation in the adult.

The *ddx-23* gene encodes a member of the evolutionarily conserved DEAD-box helicase protein family (Supplementary Fig. 1e). DEAD-box proteins are generally involved in RNA biogenesis and in humans the DEAD-box protein is a component of the spliceosomal U5 snRNP complex. In *C. elegans*, DDX-23 has been shown to regulate tissue differentiation^27^ and support microRNA biogenesis^28^. Null mutations of *ddx-23* cause embryonic lethality^28^. The two mutations we isolated with phenotypic consequences are missense mutations (G293E and I383F) of amino acid residues that are highly conserved among DDX-23 homologs (Fig. 2d, e) and likely cause a partial loss of the *ddx-23* gene function. We examined DDX-23 expression by generating a transgenic strain that contains the translational reporter *n6092 [tagRFP-T::ddx-23]*, which tags the endogenous *ddx-23* gene locus with *tagRFP-T*. tagRFP-T::DDX-23 was expressed at all stages of development and broadly in the nuclei of multiple tissues throughout the animal, including the hypodermal cells and seam cells (Supplementary Fig. 2e).

To ascertain if human DDX23 functions similarly to *C. elegans* DDX-23, we asked if the expression of a transgene encoding human DDX23 (codon-optimized for *C. elegans* expression), *ceDDX23*, could rescue the low *col-19* expression phenotype of *ddx-23* mutants. Since the brood size of *ddx-23* mutants are small and transgenic lines were difficult to establish, we scored F1 progeny from the injections as well as those transgenic line that we were able to obtain. All of the 20 F1 progeny (i.e., positive for the co-injection marker) obtained from the injection of *ddx-23* mutants with *ceDDX23* had full or partially rescued *col-19::GFP* expression, and the transgenic lines established from this experiment also partially rescued the low *col-19::GFP* expression in *ddx-23* mutants (Fig. 2f and Supplementary Fig. 1f). This result indicates that the *C. elegans ddx-23* and human *DDX23* genes likely function similarly and act in similar molecular genetic pathways, highlighting the evolutionarily conserved nature of *ddx-23* in stem-cell fate determination.

Because *col-19* encodes an adult-specific collagen and low *col-19* expression in *ddx-23* mutants (Fig. 2c) suggests a defect in the larval-to-adult transition, we asked if partial loss-of-function *ddx-23* mutants are defective in other aspects of the onset of adulthood. We first examined the formation of the adult lateral alae, distinct longitudinal cuticular structures, using electron microscopy. *ddx-23* mutants had partially disrupted alae (Supplementary Fig. 2f), consistent with the previous finding that the disruption of *col-19* function leads to defects in adult-specific alae structure^29^. Furthermore, like *mab-10* mutants, *ddx-23* mutants had seam cells that underwent extra divisions, and a *ddx-23* mutation enhanced the supernumerary-seam cell phenotype of *mab-10* mutants (Fig. 2g). In addition, in *ddx-23* mutant adults some seam cells failed to undergo the terminal fusion characteristic of the larval-to-adult transition of wild-type animals (Supplementary Fig. 2g). Like *ddx-23* single mutants, *mab-10 ddx-23* double mutants also showed a defect in seam cell fusion. *mab-10* does not function in this terminal fusion process^20^. We conclude that *ddx-23* plays multiple roles in the heterochronic pathway and the larval-to-adult transition, working with or in parallel to *mab-10* to control seam cell exit from the cell cycle while also functioning independently of *mab-10* in regulating seam cell fusion and adult alae formation (Fig. 2h and Supplementary Fig. 1a).

### DDX-23 controls MAB-10 nuclear foci formation

**Fig. 3:**
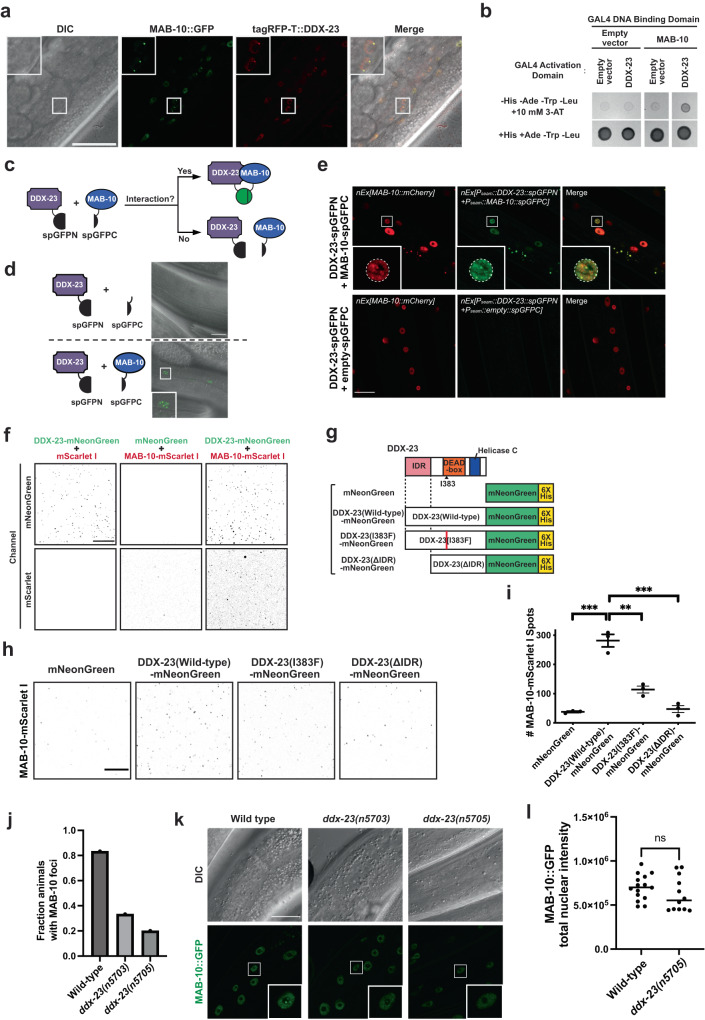
DDX-23 enhances the partitioning of MAB-10 proteins into nuclear foci. **a** Representative confocal fluorescent images of 4 animals expressing *n5909*[MAB-10::GFP] (green channel) and *n6092[tagRFP-T::ddx-23]* (red channel), which fluorescently tag *mab-10* and *ddx-23*, respectively, at their endogenous loci. Inset: Hypodermal nuclei. Scale bar, 40 μm. **b** Yeast two-hybrid spot assay showing the interaction of DDX-23 protein fused to the GAL4 activation domain (upper right spot) but not of a control with the GAL4 activation domain-only (“Empty vector”) protein with MAB-10 protein fused to the GAL4 DNA-binding domain in quadruple drop-out plates (-His/-Ade/-Trp/-Leu) containing the competitive inhibitor of histidine synthesis 3-AT. **c** Schematic representation of the split GFP (spGFP) approach for assessing protein-protein interactions in vivo. Animals expressed an N-terminal GFP fragment (spGFPN) fused to DDX-23 (purple) in combination with a C-terminal GFP fragment (spGFPC) fused to MAB-10 (blue). A physical interaction between the DDX-23::spGFPN and MAB-10::spGFPC proteins leads to the emission of green fluorescence (depicted as a green circle). **d** Representative confocal images (merged DIC and GFP channels) of spGFP experiments using DDX-23::spGFPN and MAB-10::spGFPC (or spGFPC by itself as a control). Images are representative of 8–10 animals per experimental condition. Scale bar, 20 μm. **e** Confocal fluorescence images of animals expressing a MAB-10 translational reporter (*P*_*mab-10*_*::mab-10::mCherry::mab-10 3’UTR*; red channel*)*, DDX-23::spGFPN and MAB-10::spGFPC (or empty spGFPC as a control; green channel). Image is representative of five animals per condition. Inset: seam cell. Dotted white circle, nuclear circumference; scale bar, 20 μm. **f** Representative images of in vitro assays testing heterotypic interactions of purified DDX-23-mNeonGreen and MAB-10-mScarlet I (right panel pairs). DDX-23-mNeonGreen can self-interact in the presence of mScarlet I alone (left panel pairs). MAB-10-mScarlet I can self-interact in the presence of mNeonGreen alone (middle panel pairs) but interaction is enhanced in the presence of DDX-23-mNeonGreen (right panel pairs). Images are representative of three independent experiments. Micrographs were color-inverted for better visualization. Scale bar, 200 μm. **g** Schematic view of control mNeonGreen protein alone, wild-type DDX-23-mNeonGreen, mutant DDX-23(I383F)-mNeonGreen, and a DDX-23 protein lacking its IDR (DDX-23(ΔIDR)-mNeonGreen) used for recombinant protein production. **h** Representative images of in vitro interaction assays of control mNeonGreen protein alone, wild-type DDX-23-mNeonGreen, mutant DDX-23(I383F)-mNeonGreen, and DDX-23(ΔIDR)-mNeonGreen, each with purified MAB-10-mScarlet I. Images are representative of three independent experiments. Micrographs were color-inverted for better visualization. Scale bar, 50 μm. **i** Quantification of the number of MAB-10 spots when mixed in heterotypic assays with an mNeonGreen alone control, wild-type DDX-23, mutant DDX-23[I383F] or DDX-23(ΔIDR) in three independent experiments. Error bars, mean value +/− SEM. ***P = 0.0004 (mNeonGreen vs. DDX-23(WT)-mNeonGreen), ***P = 0.0007 (DDX-23(WT)-mNeonGreen vs. DDX-23(ΔIDR)-mNeonGreen) and **P = 0.0024 (DDX-23(WT)-mNeonGreen vs. DDX-23[I383F]-mNeonGreen) (two-sided *t* tests). **j** Fraction of animals with discrete MAB-10 foci in 1-day-old wild-type, *ddx-23(n5703)*, and *ddx-23(n5705)* adults. *n* = 30 independent animals were scored for each genotype. All strains contain the reporter *n5909 [mab-10::gfp]*. **k** DIC and confocal fluorescent images of hypodermal cells in 1-day-old wild-type, *ddx-23(n5703)* and *ddx-23(n5705)* adults expressing the reporter *n5909 [mab-10::gfp]*. Images are representative of 12–15 animals per genotype. Scale bar, 20 μm. **l** Corrected total fluorescent intensity of MAB-10::GFP in the hypodermal nuclei of 1-day-old wild-type (*n* = 15) and *ddx-23(n5705)* (*n* = 12) adults expressing the reporter *n5909 [mab-10::gfp]*. ns not significant, *P* = 0.2381 (two-sided *t* test). Source data for (**i**, **j**, **l**) are provided as a Source Data file.

To examine how DDX-23 and MAB-10 function together to control seam cell exit from the cell cycle, we first tested the localization of DDX-23 and MAB-10 in vivo by using a transgenic strain that co-expresses MAB-10::GFP and tagRFP-T::DDX-23, both of which are fluorescently tagged at their endogenous locus. We observed that both MAB-10::GFP and tagRFP-T::DDX-23 are expressed in the nuclei of hypodermal cells and seam cells and that they colocalize to nuclear foci (Fig. 3a). We confirmed that the *C. elegans* MAB-10 protein can physically interact with DDX-23 in yeast two-hybrid spot assays. We observed interactions between MAB-10 and DDX-23 (Fig. 3b). We further examined the interaction between DDX-23 and MAB-10 in vivo in *C. elegans* using a split GFP (spGFP) system^30,31^ (Fig. 3c). We tagged DDX-23 with the N-terminal fragment of GFP (spGFPN) and MAB-10 with the C-terminal portion of GFP (spGFPC) and drove the expression of both by the seam cell promoter *P*_*ceh-16*_. GFP fluorescent signal should be detected only when the proteins containing the two spGFP fragments physically interact with each other. We found that DDX-23 interacted with MAB-10 in vivo in living *C. elegans* (Fig. 3d); no GFP signal was observed when an empty spGFPC control tag was used. This spGFP experiment also allowed us to identify the subcellular locations where DDX-23 and MAB-10 proteins interact. We observed diffuse spGFP signal in the seam cell nuclei along with distinct nuclear foci with concentrated spGFP fluorescence within these nuclei (Fig. 3d, inset). We then asked if these nuclear foci at which DDX-23 and MAB-10 interact are the same sites at which MAB-10 condensates form. We generated a transgenic reporter strain that expresses an *mCherry* translational reporter for MAB-10 (*P*_*mab-10*_*::mab-10::mCherry*) as well as the spGFP constructs that we used to observe the interaction between DDX-23 and MAB-10 (DDX-23-spGFPN and MAB-10-spGFPC). We showed by co-localization of the mCherry and GFP signals that the MAB-10 nuclear foci are indeed the sites of interaction between DDX-23 and MAB-10 (Fig. 3e). We note that the addition of the *mCherry* translational reporter for MAB-10 (*P*_*mab-10*_*::mab-10::mCherry*) to the spGFP experiment reduced the brightness and distinct nature of the DDX-23-MAB-10 spGFP foci; the overexpressed MAB-10::mCherry from the extrachromosomal array might have competed with MAB-10::spGFPC for the binding to DDX-23::spGFPN. Together, these data support the conclusion that MAB-10 and DDX-23 proteins interact at nuclear foci.

Both DDX-23 and MAB-10 proteins contain large intrinsically disordered regions (IDRs) (Supplementary Fig. 3a). Interactions between IDRs can act as a driving force for protein condensation into sub-nuclear bodies^32^. We tested if DDX-23 and MAB-10 can form homotypic interactions in vitro and found that both purified recombinant DDX-23-mNeonGreen fusion protein (Supplementary Fig. 3b–e) and MAB-10-mScarlet I fusion protein (Supplementary Fig. 3f–h) showed homotypic interactions. DDX-23 formed homotypic interactions readily, while MAB-10 was less efficient at forming MAB-10 spots in an homotypic assay as indicated by the quantification of fluorescent spots (Supplementary Fig. 3e, h).

Since our in vivo data showed that DDX-23 and MAB-10 proteins interact at nuclear foci (Fig. 3c–e), we next asked whether the DDX-23 protein influences the incorporation of MAB-10 protein into these nuclear structures. We assessed the in vitro formation of MAB-10–mScarlet I protein condensates in the presence or absence of a DDX-23–mNeonGreen fusion protein. The MAB-10 protein condensed more efficiently (~3.5-fold, *P* < 0.001) in the presence of DDX-23 (Fig. 3f–i and Supplementary Fig. 3i), indicating that DDX-23 can facilitate the condensation of MAB-10 protein. As shown above, the DDX-23 missense mutation I383F causes a defect in seam cell fate (Fig. 2 and Supplementary Fig. 2). We asked if this mutation can influence MAB-10 foci formation by testing the ability of MAB-10 to be incorporated into MAB-10 structures in a heterotypic assay with DDX-23[I383F] protein. The efficiency of MAB-10 incorporation into MAB-10 structures in vitro was reduced by ~60% (*P* = 0.0024) in the presence of mutant DDX-23[I383F] protein as compared to wild-type protein (Fig. 3g–i). We next asked how the IDR of DDX-23 might influence MAB-10 condensate formation. Specifically, using heterotypic assays of MAB-10 and DDX-23 proteins, we observed a 76% reduction (*P* < 0.01) in the number of MAB-10 structures formed in assays involving DDX-23 lacking its IDR (DDX-23(ΔIDR), Fig. 3g) compared to the number formed in assays performed with wild-type DDX-23 (Fig. 3h, i). These results suggest that in addition to the DEAD-box domain the low-complexity IDR of DDX-23 is also involved in driving the condensation of MAB-10.

We examined the ability of DDX-23 to facilitate the formation of MAB-10 foci in vivo by comparing MAB-10::GFP foci in wild-type and *ddx-23* mutants. We found that for the *n5703* and *n5705* alleles of *ddx-23*, the number of animals with MAB-10::GFP foci in adult hypodermal nuclei was reduced by 60% (*P* = 0.0002) and 76% (*P* < 0.0001), respectively (*n* = 30 for each genotype) (Fig. 3j, k) while the total nuclear intensity of MAB-10::GFP remained largely unchanged (Fig. 3l). This result indicates that wild-type DDX-23 protein functions in vivo to facilitate MAB-10 condensate formation in the nuclei of hypodermal and seam cells in adult animals.

### LIN-29 and MAB-10 colocalize in repressive condensates and silence larval genes, including *hedgehog*-related genes

**Fig. 4:**
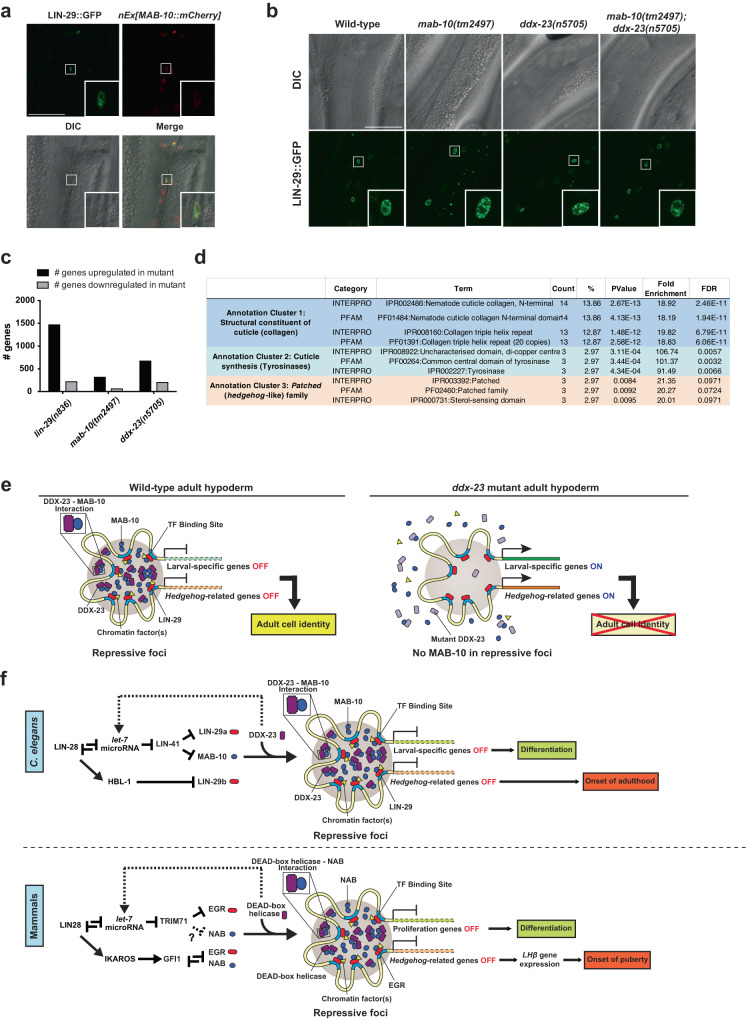
DDX-23-driven formation of MAB-10 (NAB) nuclear foci might control terminal differentiation and the onset of adulthood by transcriptionally repressing LIN-29 (EGR) target *hedgehog* and larval-specific genes. **a** Representative confocal fluorescent images of five animals expressing *n5908[lin-29::gfp]* (green channel), which fluorescently tags *lin-29* at its endogenous locus, and *nEx3004[MAB-10::mCherry]* (red channel). Inset: Hypodermal seam cell nuclei. Scale bar, 40 μm. **b** DIC and confocal fluorescent images of hypodermal cells in 1-day-old wild-type, *mab-10(tm2497), ddx-23(n5705)* and *mab-10(tm2497); ddx-23(n5705)* adults expressing the reporter *n5908[lin-29::gfp]*. Images are representative of 5-8 animals per genotype. Scale bar, 40 μm. **c** RNA-Seq analysis of *lin-29*, *mab-10*, and *ddx-23* mutant adults. The number of genes upregulated (black) and downregulated (gray) relative to wild-type adults are shown for the indicated genotypes. **d** Protein domain enrichment analysis of genes that are upregulated in *lin-29*, *mab-10*, and *ddx-23* mutant adults, as identified by RNA-Seq analysis. Statistical tests were performed according to DAVID functional annotation tools^76,77^, where Fisher’s exact test was used to determine *P* values and false discovery rates were calculated by the Benjamini–Hochberg procedure to correct for multiple testing. **e** A proposed model for how DDX-23-driven formation of MAB-10 nuclear foci controls LIN-29/MAB-10 co-regulated target gene repression in the adult hypoderm. DDX-23 binds to and enhances the partitioning of MAB-10 proteins into repressive foci, causing silencing of LIN-29/MAB-10 co-repressed target genes—including larval-specific and *Hedgehog*-related genes—and thereby defining adult cell identity (left panel). Loss-of-function mutations in *ddx-23* reduce MAB-10 partitioning into LIN-29 repressive foci and upregulate LIN-29/MAB-10 co-repressed genes including larval-specific and *Hedgehog*-related genes, resulting in a failure to maintain adult cell identity (right panel). Color key for proteins are as follows: DDX-23 (purple), MAB-10 (blue), LIN-29 (red), chromatin factor (yellow). **f** Models for how the heterochronic pathway regulates cell differentiation and the juvenile-to-adult transition in *C. elegans* (upper panel) and mammals (lower panel). For example, LIN28 signaling in mammals (LIN-28 signaling in *C*. elegans) coordinates the expression of EGR (*C. elegans* LIN-29) and NAB (*C. elegans* MAB-10) proteins, both *let-7* microRNA-dependently via TRIM71 (mammalian homolog of the *C. elegans* LIN-41) and *let-*7-independently via IKAROS (mammalian homolog of the *C. elegans* HBL-1) and GFI1. The formation of nuclear foci that contain NAB (MAB-10) proteins is facilitated by a DEAD-box helicase protein (DDX-23) and function to transcriptionally repress EGR (LIN-29) target genes including proliferation-related genes and *hedgehog*-related genes. The repression of proliferation-related genes leads to cellular differentiation, while *Hedgehog* signaling leads to luteinizing hormone LHβ expression and the onset of puberty. DDX-23 was previously described to play a role in the primary microRNA processing of *let-7* microRNA^28^ (dotted lines), possibly providing a feedback regulatory loop in this pathway. Source data for (**c**) is provided as a Source Data file.

Since MAB-10 is a co-factor for the transcription factor LIN-29 and co-regulates genes involved in terminal differentiation, we examined the relationship between LIN-29 expression and the timing of the appearance of MAB-10 foci. We generated and examined a transgenic strain that contains the translational reporter *n5908 [lin-29::gfp]*, which tags the endogenous *lin-29* locus with GFP, and an *mCherry* translational reporter for MAB-10 (*P*_*mab-10*_*::mab-10::mCherry*). We found that LIN-29 and MAB-10 proteins colocalized at nuclear foci in the hypodermal seam cells of adult animals (Fig. 4a). We next asked whether LIN-29 foci formation is dependent on MAB-10 or DDX-23 proteins. We examined the expression of LIN-29::GFP in *mab-10*, *ddx-23*, or *mab-10; ddx-23* double mutants and showed that LIN-29 foci were formed in the adult hypoderm in a MAB-10- and DDX-23-independent manner (Fig. 4b).

We next examined the roles of LIN-29, MAB-10 and DDX-23 in regulating gene expression. Since MAB-10 functions as a transcriptional co-factor for LIN-29 to regulate a subset of LIN-29 target genes and our data showed that adult-specific MAB-10 nuclear foci formation was in part facilitated by DDX-23, we performed RNA sequencing (RNA-Seq) studies of *lin-29*, *mab-10*, and *ddx-23* mutant adults and compared their gene expression profiles to that of wild-type adults. We found that the majority of genes that were misregulated in all three mutants were upregulated (Fig. 4c), showing a major role for LIN-29, MAB-10, and DDX-23 proteins in transcriptional repression. We observed a significant overlap among the genes that are upregulated in *lin-29*, *mab-10*, and *ddx-23* mutant adult animals (Supplementary Fig. 4a). This overlap was absent among the genes that were downregulated in *lin-29*, *mab-10*, and *ddx-23* mutant adult animals. These findings suggest that LIN-29, MAB-10, and DDX-23 repress a common set of target genes. We performed protein-domain enrichment analysis (Fig. 4d) and gene-enrichment analysis (Supplementary Fig. 4c) to identify categories of genes co-regulated by these three proteins. We observed that genes involved in structural constituents of the cuticle and the *Patched* (*hedgehog*-like) family of genes are enriched among the shared transcriptionally repressed targets of LIN-29, MAB-10 and DDX-23 (Fig. 4d). For example, there are many larval-specific collagen genes normally expressed highly in L4-stage larvae and downregulated in adults; these genes were upregulated in *lin-29*, *mab-10*, and *ddx-23* mutant adults compared to wild-type adults (Supplementary Fig. 4d). This observation indicates that the LIN-29, MAB-10, and DDX-23 proteins are critical in establishing the normal adult gene-expression profile and that they act by transcriptionally repressing larval-related genes.

We also found several *hedgehog*-related genes and genes that encode putative *hedgehog* receptors to be upregulated in *lin-29, mab-10* and *ddx-23* mutants (Fig. 4d and Supplementary Fig. 4e). These genes are the evolutionary homologs of *Hedgehog (Hh)* and its receptor *Patched* (*Ptc*), which control various developmental processes in species ranging from *Drosophila* to humans^33^ and have been implicated in the regulation of mammalian stem cell maintenance and proliferation^34,35^. *Hedgehog*- and *Patched*-related genes are known to be involved in *C. elegans* molting^36^ but have not been previously linked to the heterochronic pathway, which includes *lin-29, mab-10* and now *ddx-23*.

Our data suggest that LIN-29 and MAB-10 act in the same transcriptional condensate to co-repress a subset of larval-specific genes. While DDX-23 functions to concentrate MAB-10 into these foci, partitioning of LIN-29 into these foci is not dependent on MAB-10 or DDX-23. We suggest that DDX-23 binds to and facilitates the partitioning of MAB-10 protein into LIN-29 transcriptional condensates in adult hypodermal nuclei, thereby enabling MAB-10 to function as a transcriptional co-factor for LIN-29 and co-repress their target genes, which include larval genes and *hedgehog*-related genes (Fig. 4e).

## Discussion

Recent studies of transcriptional condensates have revealed new possible mechanisms of transcriptional control. There is growing evidence that transcriptional condensates are involved in both the activation^37–44^ and the repression^45–48^ of gene expression. However, little is known about how the formation of such condensates is controlled. In this study, we report that in vivo the *C. elegans* DEAD-box helicase protein DDX-23 regulates stem-cell fate at least in part by binding to and enhancing the partitioning of MAB-10 proteins into condensates, which in turn repress LIN-29/MAB-10 co-regulated target genes—including known larval-specific genes and *Hedgehog*-related genes—and thereby drive terminal cell differentiation and the onset of adulthood (Fig. 4e). Partial loss-of-function mutations in *ddx-23* reduce MAB-10 partitioning into condensates and upregulate larval-specific genes and *Hedgehog*-related genes, resulting in a failure of the stem cell-like seam cells to terminally differentiate and transition to adulthood (Fig. 4e).

Previous studies of *C. elegans*, mice and human cell lines have shown that suppression of LIN-28 levels promotes the expression of LIN-29 (EGR) and MAB-10 (NAB) proteins, both *let-7* microRNA-dependently via LIN-41 (mammalian homolog TRIM71)^16–18^ and *let-*7-independently via HBL-1 (mammalian homolog IKAROS)^18,49–51^ and GFI1^52^ (Fig. 4f). The *ddx-23* gene was previously found to play a role in the primary microRNA processing of *let-7* microRNA^28^. Here we describe a gene regulatory mechanism that regulates the activities of MAB-10 (NAB) and LIN-29 (EGR): DDX-23 drives the condensation of MAB-10 (NAB) protein into nuclear focal structures to spatiotemporally organize the nucleus and transcriptionally repress LIN-29 (EGR) target larval genes, including *hedgehog*-related genes. We propose that this process is evolutionarily conserved and that a comparable molecular genetic mechanism—in which the partitioning of NAB proteins by a DEAD-box helicase drives repression of proliferation-related and *Hedgehog*-related genes by EGR—controls mammalian stem cell development and the onset of puberty (Fig. 4f). In mammals, the DEAD-box helicase DDX5 protein^53^ and EGR1 protein^7^ act as a barrier to somatic cell reprogramming, and the DEAD-box protein Ddx20/DP103 represses transcriptional activation by Egr2^54^. We propose that DEAD-box proteins function to control EGR transcriptional activity by facilitating the condensation of its co-factor NAB, thereby coordinating molecular genetic programs that control cell differentiation.

The highly conserved *LIN28/let-7* axis has previously been found to regulate *Hedgehog* signaling in multiple contexts, e.g., during retina regeneration in zebrafish^55^ and in a mouse model for embryonal tumors with multilayered rosettes (ETMRs), a tumor-type characterized by LIN-28A overexpression^56^. *LIN28* is implicated in regulating the onset of puberty in mice and humans^9,57–61^, while *Hedgehog* signaling controls cell proliferation and cell-type determination in the pituitary gland of mice by regulating the luteinizing hormone LHβ expression, a key hormone involved in the onset of puberty^62–65^. Hence in mammals, multiple components of the evolutionarily conserved heterochronic pathway have been implicated in the onset of puberty, while in *C. elegans* this pathway similarly regulates the larval-to-adult transition. Our findings suggest a missing link between the LIN28/*let-7* axis and *Hedgehog* signaling in regulating this process: LIN28 signaling promotes the expression of EGR and NAB proteins, which in turn leads to the repression of *Hedgehog* genes. We propose that this repression is achieved by the condensation of NAB proteins to form a repressive condensate, a process driven in part by a DEAD-box helicase protein and that the repression of *Hedgehog-*related genes subsequently induces the expression of the luteinizing hormone LHβ and leads to cell differentiation and the onset of puberty (Fig. 4f).

Our findings also have implications for the understanding of molecular mechanisms of carcinogenesis. The overexpression of human *LIN28* inhibits *let-7* microRNA gene activity and thereby promotes cancerous growth^12,66^, possibly by augmenting downstream *Shh* Hedgehog signaling^56^. We speculate that this regulation is driven by the downregulation of EGR, which acts as a tumor suppressor and is commonly mutated or suppressed in multiple human cancers^67,68^ and that this downregulation leads to the upregulation of EGR target genes (including *Hedgehog* genes) that are normally silenced to regulate cell differentiation and the onset of adulthood. In healthy cells, DEAD-box helicase proteins ensure repression of EGR target genes essential to cell proliferation by facilitating the formation of MAB-10 (NAB) repressive transcriptional condensates; failure to form such condensates could lead to abnormal cell proliferation. Indeed, *DDX23* mutations and deletions have been detected in several human cancers^69^, suggesting a tumor suppressor role for this gene. Based on our findings, we propose as a mechanism that these *DDX23* genetic defects result in a failure in the formation of DDX23-NAB transcriptional condensates and in the consequent upregulation of EGR-repressed targets—which include genes involved in *Hedgehog* signaling—leading to the abnormal cell proliferation that drives cancerous growth.

## Methods

### *C. elegans* strains and transgenes

All *C. elegans* strains were cultured as described previously^70^. We used the N2 Bristol strain as the reference wild-type strain, and the polymorphic Hawaiian strain MT22656 for genetic mapping and SNP analysis^71^. All strains were maintained at 20 °C. Details about the strains used in this study can be found in Supplementary Table 1.

We used the following mutations and transgenes:

LGII: *mab-10(tm2497, n5909 [mab-10::gfp]), lin-29(n546, n836, n5908 [lin-29::gfp])*

LGIII: *ddx-23(n5703, n5705, n6026[I383F], n6027[S365N])*

LGIV: *wIs78 [ajm-1::gfp + scm::gfp]*

LGV: *maIs105[col-19::gfp]*.

### Extrachromosomal arrays

*nEx2848[P*_*ddx-23*_*::ddx-23::ddx-23 3’UTR, P*_*ttx-3*_*::mCherry::tbb-2 3’UTR, P*_*rab-3*_*::mCherry::unc-54 3’UTR], nEx2958[P*_*ddx-23*_*::ddx-23::TagRFP-T::ddx-23 3’UTR, P*_*myo-2*_*::mCherry::unc-54 3’UTR], nEx2971[P*_*ddx-23*_*::ceDDX23::ddx-23 3’UTR, P*_*myo-2*_*::mCherry::unc-54 3’UTR #1], nEx2972[P*_*ddx-23*_*::ceDDX23::ddx-23 3’UTR, P*_*myo-2*_*::mCherry::unc-54 3’UTR #2], nEx2768[P*_*ceh-16*_*::DDX-23 cDNA::spGFPN::unc-54 3’UTR, P*_*ceh-16*_*::MAB-10 cDNA::spGFPC::unc-54 3’UTR, P*_*rab-3*_*::mCherry::unc-54 3’UTR], nEx2769[P*_*ceh-16*_*::DDX-23 cDNA::spGFPN::unc-54 3’UTR, P*_*ceh-16*_*::empty::spGFPC::unc-54 3’UTR, P*_*rab-3*_*::mCherry::unc-54 3’UTR], nEx2931[P*_*mab-10*_*::MAB-10::mCherry::mab-10 3’UTR, P*_*ceh-16*_*::DDX-23 cDNA::spGFPN::unc-54 3’UTR, P*_*ceh-16*_*::MAB-10 cDNA::spGFPC::unc-54 3’UTR, P*_*myo-2*_*::mCherry::unc-54 3’UTR], nEx2934[P*_*mab-10*_*::MAB-10::mCherry::mab-10 3’UTR, P*_*ceh-16*_*::DDX-23 cDNA::spGFPN::unc-54 3’UTR, P*_*ceh-16*_*::empty::spGFPC::unc-54 3’UTR, P*_*myo-2*_*::mCherry::unc-54 3’UTR], nEx3004[P*_*mab-10*_*::MAB-10::mCherry::mab-10 3’UTR, P*_*myo-2*_*::mCherry::unc-54 3’UTR]*.

### Molecular cloning and transgenic strain construction

To endogenously tag the *mab-10* genomic locus at its C-terminus with GFP (transgene *n5909[mab-10::gfp])* using CRISPR-Cas9^72^, we generated a *mab-10::gfp* homologous repair template for Cas9-mediated *gfp* knock-in. We PCR-amplified a ~3 kb genomic region centered on the *mab-10* stop codon from N2 genomic DNA and cloned the resulting fragment into the pCR-Blunt vector using the ZeroBlunt TOPO Cloning Kit (Thermo Fisher). Using the Gibson assembly kit (New England Biolabs), we inserted a GA-linker and *gfp* in-frame between the last amino acid codon of the *mab-10* gene and the stop codon, resulting in a homologous repair template with ~1.5 kb homology arms. To avoid cleavage of the repair template by Cas9, we mutated the PAM motif of the Cas9 target site in the repair template. We also generated a Cas9-sgRNA plasmid to cleave upstream of the *mab-10* stop codon by inserting a targeting sequence (5’-GAAAAAGTAGCAATACAACA-3’) into the pDD162 Cas9-sgRNA plasmid^72^ by site-directed mutagenesis. Forward primer 5’-GAAAAAGTAGCAATACAACAGTTTTAGAGCTAGAAATAGCAAGT-3’ and reverse primer 5’-CAAGACATCTCGCAATAGG-3’ were used. We co-injected the homologous repair template, the *mab-10* Cas9-sgRNA plasmid, and an *unc-22* sgRNA co-CRISPR plasmid into wild-type N2 worms. Twitching progeny (indicative of successful *unc-22* co-CRISPR integration) were selected and genotyped for integration of *gfp* into the *mab-10* locus. Finally, the strain was backcrossed to wild-type N2 animals to remove the *unc-22* mutation.

To create the *ddx-23(n6026)* allele encoding DDX-23[I383F] and the *ddx-23(n6027)* allele encoding DDX-23[S365N] by CRISPR-Cas9, we used oligonucleotides as repair templates to introduce substitutions as previously described^73^. pDD162 was used as a template for site-directed mutagenesis to insert Cas9 targeting sequences for *ddx-23(n6026)*(5’-ACGACAAGAACATCGGGACT-3’) and *ddx-23(n6027)*(5’-AATGGAACGACAAGAACATC-3’).Temperature-sensitive *pha-1* co-conversion was performed as previously described^73^.

To generate the *ddx-23* rescuing transgene *nEx2848*, the region spanning 5 kb upstream of the *ddx-23* gene (*P*_*ddx-23*_), *ddx-23* genomic DNA, and the 3’UTR were PCR-amplified using forward primer 5’-GGTACAACCATGTTCAAATCAGCATCC-3’ and reverse primer 5’-CGTGAGTTTGGTTCTGGAGCTT-3’. This PCR fragment was cloned into a pUC19 vector and a Tag-RFP-T sequence was inserted immediately before the *ddx-23* stop codon to generate the *ddx-23* rescuing transgene *nEx2958. P*_*ddx-23*_*::ceDDX23::ddx-23 3’UTR (nEx2971* and *nEx2972)* was generated by HiFi DNA Assembly (New England Biolabs) of *P*_*ddx-23*_, wild-type human DDX23 cDNA codon-optimized for expression in *C. elegans* and containing 3 synthetic introns generated using gBlocks Gene Fragments (Integrated DNA Technologies), and the *ddx-23* 3’UTR in a pUC19 vector. For the split-GFP protein-protein in vivo interaction assays, all cloning was performed using the HiFi DNA Assembly system. The N-terminal GFP (spGFPN) plasmid was cloned by inserting the *ddx-23* cDNA and spGFPN fragment between a 2.5 kb *ceh-16* promoter and the *unc-54* 3’UTR. The C-terminal GFP (spGFPC) plasmid was cloned by inserting the *mab-10* cDNA (or an ATG start codon sequence for the “empty” control) and the spGFPC fragment between a 2.5 kb *ceh-16* promoter and the *unc-54* 3’UTR. Complete plasmid sequences of all plasmids are available from the authors upon request.

Transgenic strains were generated by germline transformation as described^74^. All transgenic constructs were injected at 1–50 ng/ml.

### Microscopy and image analysis

All *C. elegans* micrographs excluding electron micrographs are of lateral views of hermaphrodite animals. Nomarski DIC and epifluorescence micrographs were obtained using a Zeiss Axio Imager Z2 compound microscope and Zen Blue (Zeiss) software. Confocal microscopy was performed using the Zeiss LSM 800 instrument, and images were obtained using the Zen Blue 2.0 (Zeiss) software. The resulting images were prepared using ImageJ (National Institutes of Health) and Adobe Illustrator CS4 softwares. Image acquisition settings were calibrated to minimize the number of saturated pixels and were kept constant throughout each experiment. Graphs and indicated statistical analyses were performed using GraphPad Prism 6 software. For electron micrographs, animals were assayed by picking L4 males to plates, fixing 24 h later, and performing electron microscopic analysis, as previously described.

### Fluorescence recovery after photobleaching (FRAP)

We immobilized 1-day-old adult animals with 10 mM levamisole (an acetylcholine agonist that induces muscle contraction) on 2% agarose pads for confocal imaging using the Zeiss LSM 800. We imaged hypodermal cells that were proximal to the cover slip. We identified two imaging regions of interest (ROIs) for each FRAP experiment (one for FRAP measurements, one for an imaging-associated bleaching control). We manually defined the FRAP ROI as the region around a given MAB-10::GFP focus. As a control, we performed FRAP studies of the diffuse MAB-10::GFP signal in hypodermal cells. To achieve complete bleaching of GFP in the FRAP ROI, bleaching was conducted with 25 rounds of 100% laser induction with a 488 nm laser. Hypodermal cells used to perform FRAP studies of diffuse MAB-10::GFP signals (“Diffuse”) were then tracked for at least 35 s, while hypodermal cells used to perform FRAP studies of MAB-10::GFP foci (“Focus”) were tracked for at least 80 s. Data were analyzed using ImageJ, measuring the fluorescence intensity for all frames and fitting the FRAP curve by an increasing exponential.

### Mutagenesis screen for enhancers of *mab-10* and mapping of *ddx-23*

To screen for enhancers of the *mab-10* seam cell defect, we mutagenized *mab-10(tm2497)* mutants with ethyl methanesulfonate (EMS) as described previously^70^. The starting strain contained the *col-19::gfp* (*maIs105*) transgene, which shows a reduced expression level in *mab-10(lf)* adults relative to that of wild-type adults and served as a reporter for the heterochronic pathway. We used a Nikon SMZ18 fluorescence dissecting microscope to screen F2 progeny of mutagenized hermaphrodites for second-site mutations that further reduced the low hypodermal *col-19* expression levels of *mab-10(tm2497)* mutants. Screen isolates were backcrossed to an otherwise wild-type strain containing the *maIs105* reporter. *ddx-23(n5703)* and *ddx-23(n5705)* were identified by crossing the mutant isolate with a Hawaiian strain containing the *maIs105* reporter, isolating the F2 progeny with low hypodermal *col-19* expression and mapping the mutation using single-nucleotide polymorphisms^71^ and whole-genome sequencing. Transgenic rescue experiments demonstrated that these *ddx-23* mutations were the causative mutations, as described in the text.

### Seam cell count

The number of seam cells was quantified in 1-day-old adults, using either the *maIs105[col-19::gfp]* or *wIs78[ajm-1::gfp + scm::gfp]* transgenes. DIC and epifluorescence optics using a Zeiss 63x objective lens on a Zeiss Axio Imager Z2 compound microscope were used. The number of seam cells was scored by counting GFP-positive seam cell nuclei along the length of each animal.

### Yeast two-hybrid binding assay

*ddx-23* and *mab-10* cDNAs were cloned into pGADT7 and pGBKT7 plasmids (Takara), and the constructs were co-transformed into yeast strain Y2HGold (Takara). Co-transformants were selected on SD plates containing minimal supplements without leucine and tryptophan (SD/-Leu/-Trp) for 2 days at 30 °C. To test for protein interaction, co-transformants were picked and grown overnight in SD/-Leu/-Trp broth. Cultures were diluted to a similar optical density at 600 nm, and 3 μl of each culture was spotted onto SD plates containing minimal supplements without tryptophan, leucine, histidine, and adenine (SD/-Leu/-Trp/-His/-Ade) and containing 10 mM of the competitive inhibitor of histidine synthesis 3-AT, and cultured for 2 days at 30 °C.

### Recombinant protein expression and protein purification

HiFi DNA Assembly into a pDEST17 (Thermo Fisher) backbone was used to create all bacterial expression vectors. For the recombinant DDX-23 expression vector, the *ddx-23* cDNA sequence followed by mNeonGreen and a 6xHis tag at its 3’ end was inserted into pDEST17. Site-directed mutagenesis of this plasmid was performed to create the DDX-23 I383F mutant and the DDX-23(ΔIDR) mutant (removing amino acid residues 2–265). For the recombinant MAB-10 expression vector, *mab-10* cDNA followed by mScarlet I and a 6xHis tag was inserted into pDEST17. Vectors expressing C-terminally 6xHis-tagged mNeonGreen and mScarlet I were created as “empty” controls.

For protein expression, plasmids were transformed into Shuffle T7 competent *E. coli* cells (New England Biolabs). A fresh bacterial colony was inoculated into LB medium containing ampicillin and grown overnight at 30 °C. Cells were diluted 1:100 in 1 liter of LB medium containing ampicillin and grown at 30 °C until the OD_600_ reached between 0.5 and 0.6. IPTG was added to 0.5 mM, followed by incubation at 16 °C for 18 h. Cells were collected and stored frozen at −80 °C.

For protein purification, bacterial pellets were resuspended in 15 ml of lysis buffer (20 mM Tris-HCl pH 7.4, 500 mM NaCl, 10 mM imidazole, 14 mM β-mercaptoethanol (ME), 10% (v/v) glycerol, 1% Triton-X) with Complete EDTA-free protease inhibitors (Roche) and 15 mg of lysozyme, and sonicated. The lysates were cleared by centrifugation at 10,000 × *g* for 30 min and added to 1 ml of Ni-NTA agarose (Qiagen) pre-equilibrated with the same lysis buffer, and rotated at 4 °C for 2 h. The slurry was centrifuged at 3000 × *g* for 3 min. The resin pellets were washed five times with 4 ml of wash buffer (20 mM Tris-HCl pH 7.4, 500 mM NaCl, 14 mM β-ME, 10% (v/v) glycerol, and 25 mM imidazole), followed by centrifugation as above. Protein was eluted twice for 15 min with 2 ml of Ni-NTA Elution Buffer (20 mM Tris pH 7.4, 500 mM NaCl, 14 mM β-ME, 10% (v/v) glycerol, and 250 mM imidazole). We further purified the DDX-23 and MAB-10 proteins using heparin columns. For DDX-23-mNeonGreen-6xHis and DDX-23[I383F]-mNeonGreen-6xHis, eluates were pooled and diluted in 20 ml of 1:1 mixture of Heparin Binding Buffer (20 mM Tris pH 7.4, 50 mM NaCl, 1% (v/v) glycerol, 2 mM DTT) and Heparin Elution Buffer (20 mM Tris, pH 7.4, 1 M NaCl, 1% (v/v) glycerol, 2 mM DTT). For DDX-23(ΔIDR)-mNeonGreen-6xHis and MAB-10-mScarlet I-6xHis, eluates were diluted in 20 ml of Heparin Binding Buffer. The protein sample was applied to a pre-equilibrated heparin column with a flow rate of 1 ml/min, followed by a column wash using the same buffer in which the protein had been diluted. DDX-23-mNeonGreen-6xHis and DDX-23[I383F]-mNeonGreen-6xHis proteins were eluted in Heparin Elution Buffer; DDX-23(ΔIDR)-mNeonGreen-6xHis protein was eluted in 7:3 ratio of Heparin Binding Buffer: Heparin Elution Buffer; MAB-10 protein was eluted with a series of step elutions with Heparin Binding Buffer: Heparin Elution Buffer ratios ranging from 3:1 to 1:1. All recombinant mNeonGreen or mScarlet I fusion proteins were concentrated using Vivaspin 500 Centrifugal Concentrators (10,000 MWCO). We analyzed the proteins using a 12% acrylamide gel followed by Coomassie staining to assess protein purity. Protein concentration was measured using the Pierce 660 nm Protein Assay Kit (Thermo Fisher).

### In vitro protein interaction assay

As described in the figure legends, recombinant proteins were added to solutions at varying concentrations with 200–220 mM final NaCl with or without 10% PEG-8000 as crowding agent in the reaction buffer (50 mM Tris-HCl pH 7.4, 10% glycerol, 1 mM DTT). The protein solution was loaded onto glass slides within a silicone isolator (Sigma) and imaged using a Zeiss LSM 800. For all experiments, at least three independent mixtures were analyzed. For better visualization, micrographs were color-inverted using ImageJ for Fig. 3f, h. For quantification of fluorescent spots, we performed Particle Analysis using ImageJ with a size threshold of 1 μm^2^.

### Gene expression analyses

For RNA-Seq experiments, total RNA from three biological replicates of age-synchronized young adult hermaphrodites (200 in total, picked manually) of wild-type, *lin-29(n836), mab-10(tm2497), and ddx-23(n5705)* genotypes were prepared using a BeadBug microtube homogenizer (Sigma) and 0.5-mm zirconium beads (Sigma). RNA was extracted using the RNeasy Mini kit (QIAGEN) according to the manufacturer’s instructions. RNA libraries were prepared for sequencing using standard Illumina protocols and the NeoPrep system. RNA-Seq was performed using the Illumina NextSeq500 platform, and data were analyzed using standard protocols^75^. Reads were mapped against *Caenorhabditis elegans* ws258 reference sequences using STAR/2.5.3a, and gene expression was quantified using RSEM v. 1.3.0. Differential gene expression analysis was performed using DESeq2. An adjusted *P* value cutoff of <0.05 and a fold change of more than 2 was used to identify differentially expressed genes between the wild-type and mutants. There were 102 genes—including larval-specific collagen genes and several *hedgehog*-related (*grd)* genes and putative *hedgehog* receptor (*ptr*) genes—that were upregulated in all three mutants (*lin-29, mab-10*, and *ddx-23* mutants) relative to wild-type animals. We performed gene ontology enrichment analysis and protein domain enrichment analysis using DAVID functional annotation tools^76,77^.

### Statistical analysis

Unpaired, two-tailed *t* tests were used to compare half-maximal recovery times from FRAP studies of MAB-10::GFP in foci and diffuse signals, numbers of seam cells that undergo extra seam cell division between strains, normalized gene expression abundance log_2_(fpkm+1) of multiple genes between strains, the number of homotypic droplets of control mNeonGreen alone to that of purified DDX-23-mNeonGreen, the number of homotypic droplets of control mScarlet I alone to that of purified MAB-10-mScarlet I, and the number of MAB-10 droplets in mixed heterotypic assays with control, wild-type DDX-23, mutant DDX-23[I383F] or DDX-23(ΔIDR) proteins. Two-tailed t-tests were also used to compare MAB-10 total nuclear intensity in wild-type and *ddx-23(n5705)* animals, and *maIs105* transgene levels in wild-type, *mab-10, ddx-23, mab-10; ddx-23* and *ddx-23(+)* rescue animals. Fisher’s exact test was used to compare the number of animals that have MAB-10::GFP foci in adult hypodermal nuclei in wild-type versus *ddx-23(n5703)* or *ddx-23(n5705)* mutants. Gene ontology enrichment analysis was performed according to DAVID functional annotation tools^76,77^; Fisher’s exact test was used to determine *P* values, and False Discovery Rates were calculated using the Benjamini–Hochberg procedure to correct for multiple testing. Statistical tests were performed using GraphPad Prism 6 software. Biological replicates were performed using separate populations of animals.

### Reporting summary

Further information on research design is available in the Nature Portfolio Reporting Summary linked to this article.

### Supplementary information

Supplementary Information

Reporting Summary

### Source data

Source Data

## Data Availability

All data required to assess the study’s conclusions are present in the main text and supplementary materials. The RNAseq dataset generated in this study has been deposited in the NCBI GEO database under accession code GSE151399. *Caenorhabditis elegans* ws258/WBcel235 reference sequences were used. Further information and requests for resources and reagents should be directed to and will be fulfilled by the Lead Contact, H. Robert Horvitz (horvitz@mit.edu). Source data are provided with this paper.
