# Supplementary material for: A DEAD-box helicase drives the partitioning of a pro-differentiation NAB protein into nuclear foci
Source: Nat Commun. 2023 Oct 18;14:6593. doi: 10.1038/s41467-023-42345-9 (PMC10584935; doi:10.1038/s41467-023-42345-9)
Supplement: Supplementary file 1 — Supplementary Information [file 41467_2023_42345_MOESM1_ESM.pdf]

Supplementary Information

Supplementary Figure 1
Supplementary Figure 2
Supplementary Figure 3
Supplementary Figure 4
Supplementary Table 1

A DEAD-box helicase drives the partitioning of a pro-differentiation NAB protein into nuclear foci

Akiko Doi[1], Gianmarco D. Suarez[1], Rita Droste[1], and H. Robert Horvitz[1,*]

[1]Howard Hughes Medical Institute, Department of Biology, Massachusetts Institute of Technology, Cambridge, MA 02139, USA

*Correspondence: horvitz@mit.edu

Supplementary Figure 1

a

	Wild type	*lin-29*	*mab-10*	*ddx-23*
Adult cuticle synthesis	+	–	+	–
Exit from molting cycle	+	–	–	+
Seam cell exit from the cell cycle	+	–	–	–
Seam cell fusion	+	–	+	–

b

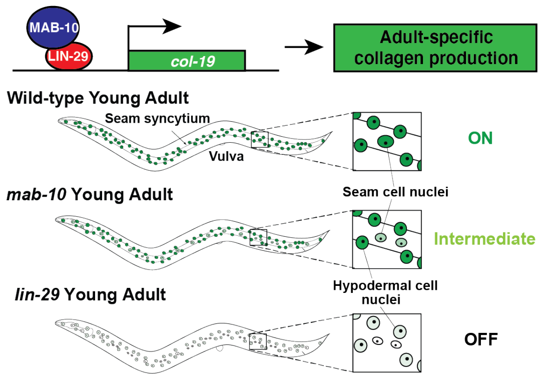

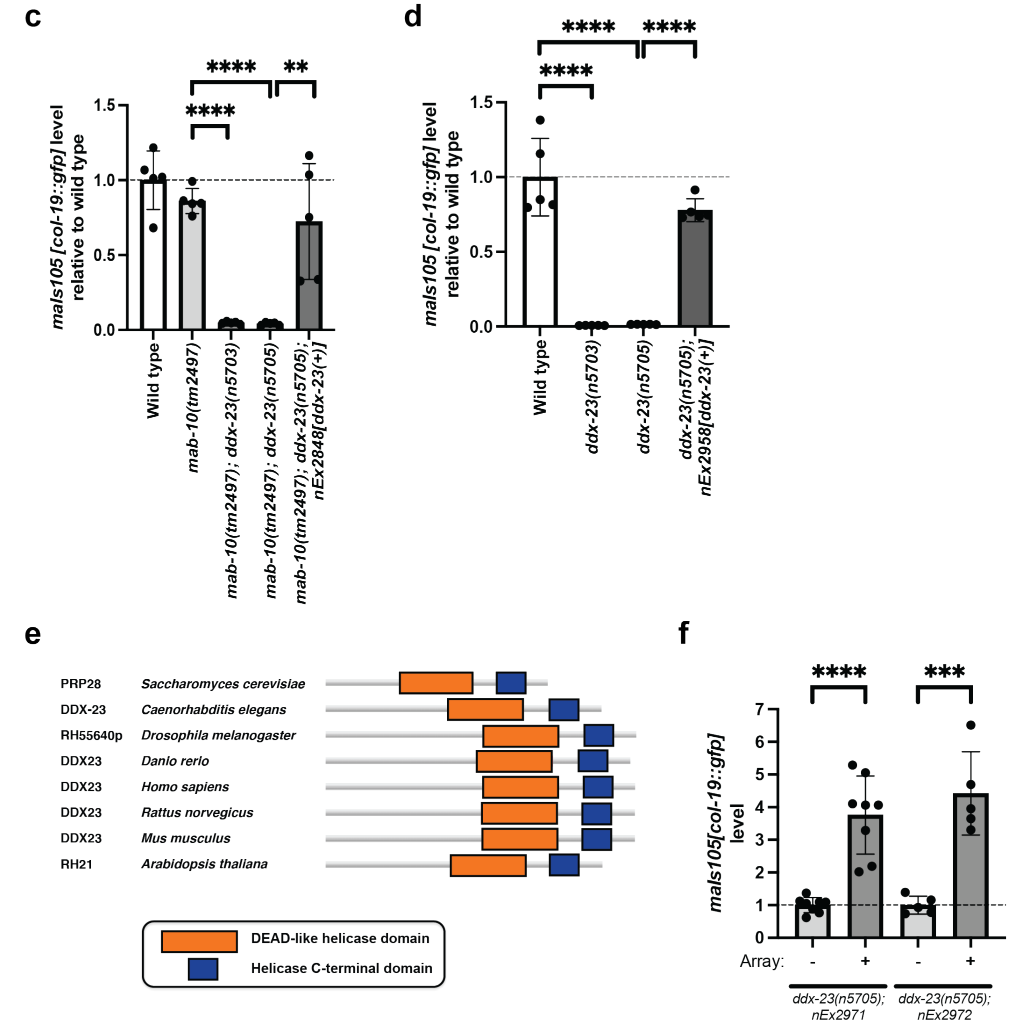

Supplementary Figure 1. *lin-29* and *mab-10* mutant phenotypes and molecular identification of *ddx-23*. a, Presence(+) or absence(-) of the four key characteristic events that normally occur during the larval-to-adult transition for the indicated genotypes. **b,** Rationale for the F2 non-clonal screen for factors that act with or in parallel to MAB-10. The *col-19::gfp (maIs105)* transgene was used as a reporter for the heterochronic pathway. In wild-type animals *maIs105* is up-regulated in adults, whereas in *lin-29* mutant adult animals this reporter is not expressed. In *mab-10* mutant adult animals there is a reduced level of *maIs105* expression. **c,** Relative reporter GFP expression of *col-19::gfp (maIs105)* in the isolates from the screen (*mab-10(tm2497); ddx-23(n5703)* and *mab-10(tm2497); ddx-23(n5705))*, and rescue of low GFP expression in the presence of an extrachromosomal array expressing *ddx-23.* GFP quantification was performed in strains shown in Fig. 2b (5 animals per genotype). Error bars, mean value +/- SD. *****p* = 2.20 x 10^{-8} (*mab-10(tm2497)* vs. *mab-10(tm2497);ddx-23(n5703)*), *p* = 2.05 x 10^{-8} (*mab-10(tm2497)* vs. *mab-10(tm2497);ddx-23(n5705)*), ***p*=0.0043 (Two-sided t-test). **d,** Relative reporter GFP expression of *col-19::gfp (maIs105)* in *ddx-23(n5703)* and *ddx-23(n5705)* single mutants, and rescue of low GFP expression in the presence of an extrachromosomal array expressing *ddx-23.* GFP quantification was performed in strains shown in Fig. 2c (5 animals per genotype). Error bars, mean value +/- SD. *****p* = 2.66 x 10^{-5} (wild-type vs. *ddx-23(n5703)*), *p* = 2.80 x 10^{-5} (wild-type vs. *ddx-23(n5705)*), *p* = 1.87 x 10^{-8} (*ddx-23(n5705)* vs. *ddx-23(n5705);nEx[ddx-23(+)]*) (Two-sided t-test). **e,** Homologs of the *C. elegans* DDX-23 protein contain the DEAD-like helicase domain (orange) and the helicase C-terminal domain (blue). **f,** Relative reporter GFP expression of *col-19::gfp (maIs105)* in *ddx-*

23(n5705) when overexpressing human *DDX23* codon-optimized for expression in *C. elegans (ceDDX23).* GFP quantification was performed in strains shown in Fig. 2f. 5-8 animals per genotype were analyzed. Error bars, mean value +/- SD. ****$p = 1.66 \times 10^{-5}$ (*ddx-23(n5705);nEx2971 +/-* array), ***p=0.0004 (*ddx-23(n5705);nEx2972 +/-* array) (Two-sided t-test).

Source data for panels **c**, **d**, and **f** are provided as a Source Data file.

Supplementary Figure 2

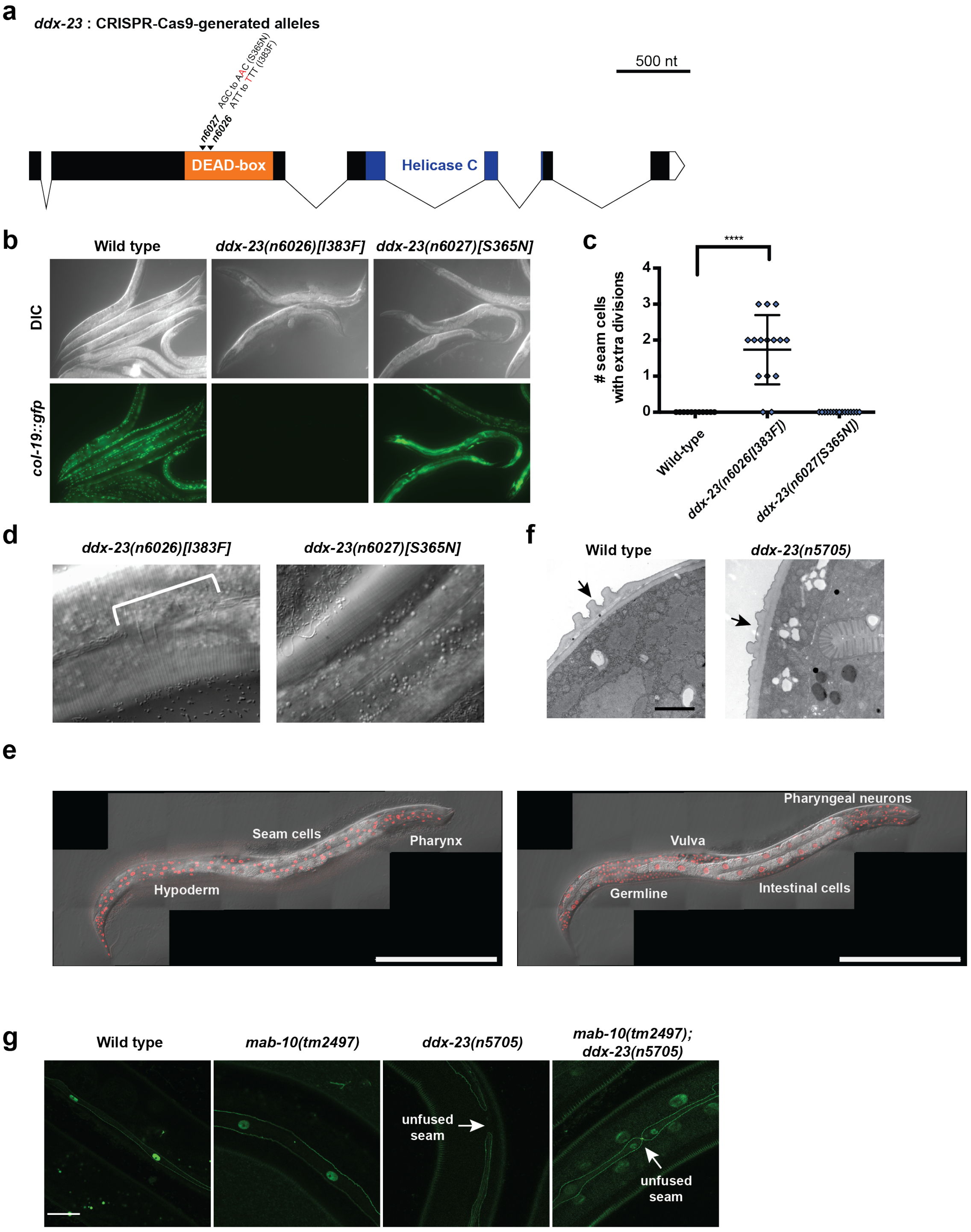

Supplementary Figure 2. CRISPR-Cas9-generated *ddx-23(n6026)* allele encoding DDX-23[I383F] phenocopies the *ddx-23(n5705)* abnormal phenotype. a, Schematic of *ddx-23* gene structure. *ddx-23(n6026)[I383F]* and *ddx-23(n6027)[S365N]* alleles were individually recreated using the CRISPR-Cas9 genome-editing technology. Both of these DDX-23 missense mutations (S365N, I383F) were present in the strain isolated from the *mab-10* enhancer screen. For simplicity, we designated the allele *ddx-23(n5705)* from the *mab-10* enhancer screen as the causal mutation for the abnormal phenotypes and concluded that the DDX-23[I383F] mutation is responsible for the phenotypic effects based on the observation that *ddx-23(n6026)[I383F]* but not *ddx-23(n6027)[S365N]* phenocopied the *ddx-23(n5705)* seam cell abnormalities (Fig. 2c, g and Supp Fig. 2b-d). **b,** Representative micrographs of *maIs105[col-19::gfp]* reporter expression in one-day old adults of the indicated genotypes. *ddx-23(n6026)[I383F]* but not *ddx-23(n6027)[S365N]* mutants showed low *col-19* transgene expression. Images are representative of 11-15 animals per genotype. **c,** Number of seam cells that undergo extra seam cell division, scored in one-day old wild-type (n = 11), *ddx-23(n6026)[I383F]* (n = 15), and *ddx-23(n6027)[S365N]* (n = 15) adults. *ddx-23(n6026)[I383F]* but not *ddx-23(n6027)[S365N]* mutants had seam cells that underwent extra divisions. Error bars, mean value +/- SD. ****$p = 3.87 \times 10^{-6}$ (Two-sided t-test). **d,** Representative micrographs of the lateral alae, longitudinal cuticular ridges generated by the seam cells, of *ddx-23(n6026)[I383F]* and *ddx-23(n6027)[S365N]* one-day old adults. *ddx-23(n6026)[I383F]* but not *ddx-23(n6027)[S365N]* mutants had defective alae formation. Images are representative of 15 animals per genotype. **e,** Representative micrographs of late L4 stage animals expressing *n6092[tagRFP-T::ddx-23]*. Images are representative of 10 animals. Two confocal microscopy planes are shown to illustrate the various cell types in which *n6092[tagRFP-T::ddx-23]* is expressed, Scale bar, 200 µm. **f,** Electron micrographs of cross-sections of wild-type and *ddx-23(n5705)* 24 hour adult males. Images are representative of 3 animals per genotype. Arrows indicate adult-specific lateral alae. Scale bars, 2 µm. **g,** Confocal fluorescence images of seam cell fusion events observed in one-day old adults of the indicated genotypes containing the reporter *wIs78[ajm-1::gfp + scm::gfp]*, which marks

the seam cell nuclei and adherens junctions. Images are representative of 5-8 animals per genotype. Scale bar, 20 μm.

Source data for panel **c** is provided as a Source Data file.

Supplementary Figure 3

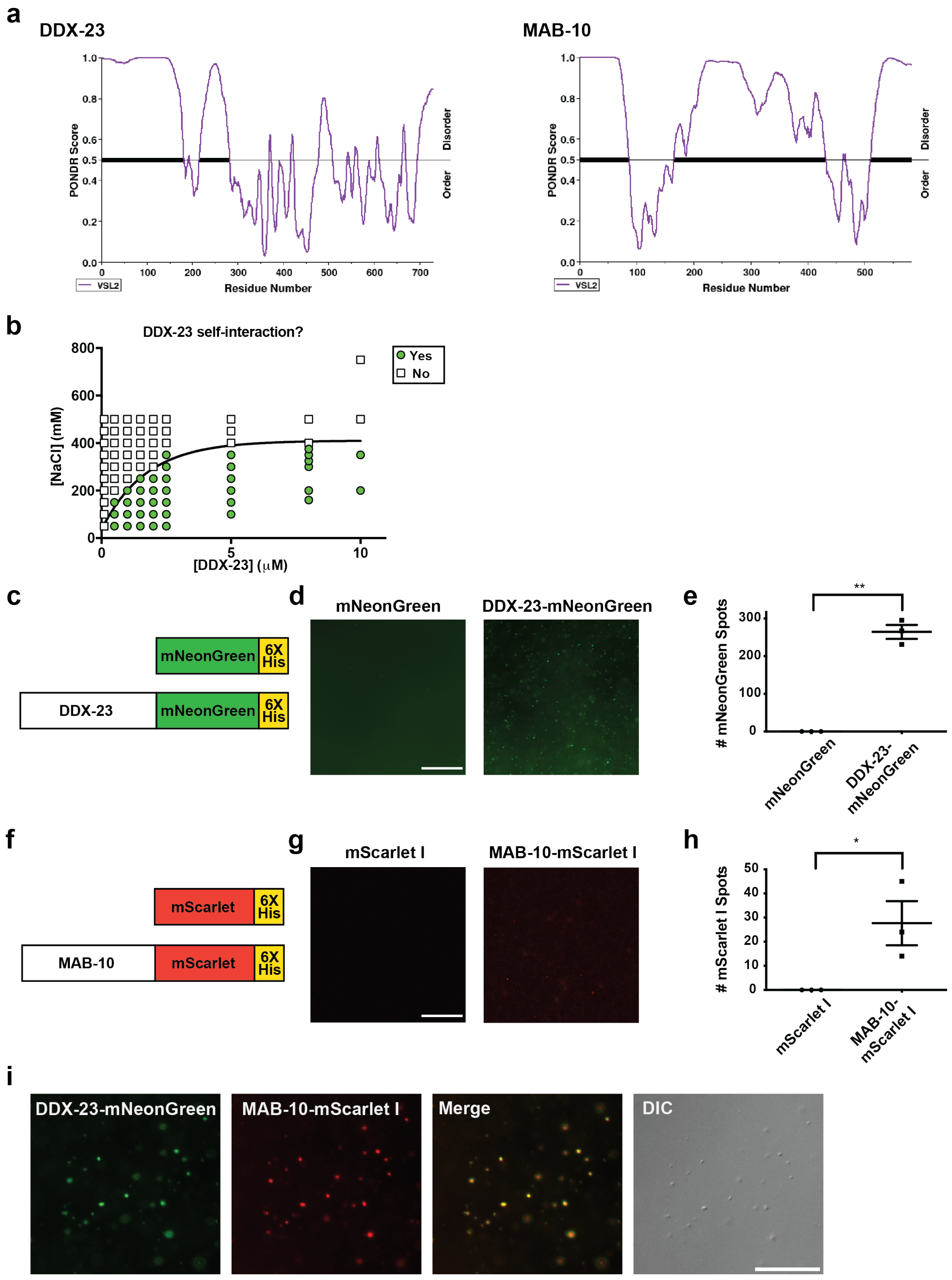

Supplementary Figure 3. DDX-23 and MAB-10 self-interact and interact with each other. a, Graphs plotting intrinsic disorder of DDX-23 (left panel) and MAB-10 (right panel). PONDR (Predictor of Natural Disordered Regions) VSL2 scores[1] (http://www.pondr.com/) are shown on the y axis, and amino acid positions are shown on the x axis. PONDR scores show predictions of ordered (PONDR score < 0.5) or disordered (PONDR score > 0.5) structure throughout the protein. **b,** Diagram plotting purified DDX-23 protein and NaCl concentrations, scoring for presence (green circles) or absence (white squares) of optically resolvable spots. Assays were performed in the absence of the molecular crowder PEG-8000[2] to determine if the DDX-23 protein is capable of self-interacting under physiologically relevant concentrations. **c,** Schematic view of control mNeonGreen protein and DDX-23-mNeonGreen used for recombinant protein production. **d,** Representative images ($n = 3$) of *in vitro* assays testing homotypic interaction of purified DDX-23-mNeonGreen (right panel) and mNeonGreen alone as a control (left panel). Assays were performed in the presence of 220 mM NaCl using 500 nM purified protein (no PEG-8000). Scale bar, 200 µm. **e,** Number of spots of control mNeonGreen alone and of DDX-23-mNeonGreen. Data from 3 independent experiments were analyzed. Error bars, mean value +/- SEM. $^{**}p = 0.001$ (Two-sided t-test). **f,** Schematic view of control mScarlet I protein and MAB-10-mScarlet I used for recombinant protein production. **g,** Representative images ($n = 3$) of *in vitro* assays testing homotypic interaction of purified MAB-10-mScarlet I (right panel) and mScarlet I alone as a control (left panel). Assays were performed in the presence of 220 mM NaCl using 500 nM purified protein (no PEG-8000). Scale bar, 200 µm. **h,** Number of homotypic spots of control mScarlet I alone and of MAB-10-mScarlet I. Data from 3 independent experiments were analyzed. Error bars, mean value +/- SEM. $^{*}p = 0.0388$ (Two-sided t-test). **i,** Representative micrograph of *in vitro* assays testing heterotypic interactions between purified DDX-23-mNeonGreen and MAB-10-mScarlet I. Scale bar, 50 µm.

Source data for panels **b**, **e**, and **h** are provided as a Source Data file.

Supplementary Figure 4

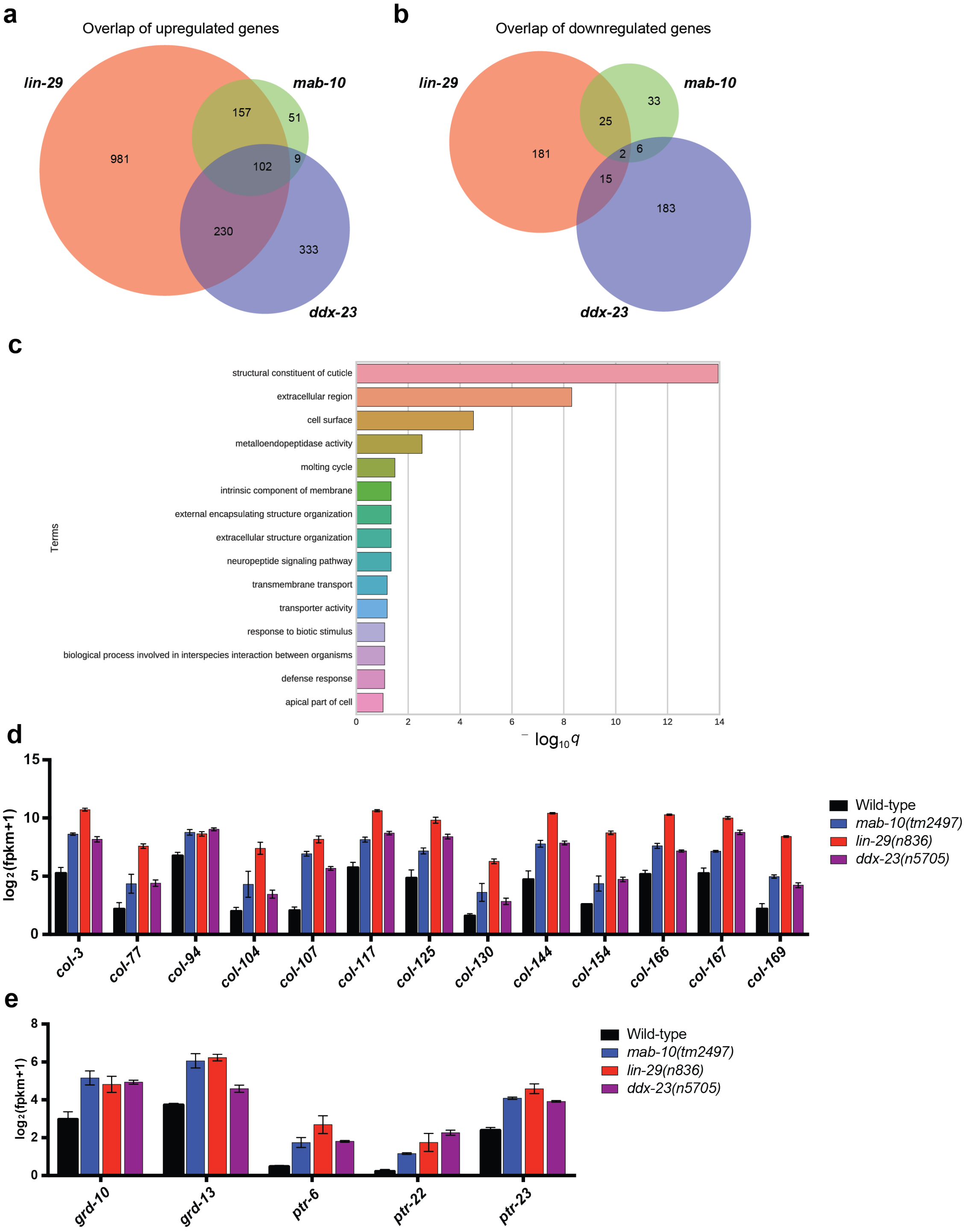

Supplementary Figure 4. LIN-29, MAB-10, and DDX-23 proteins are critical in establishing an adult gene expression profile by transcriptionally repressing larval-related genes and *hedgehog*-related genes. a, Venn diagram depicting the

overlap in genes upregulated in *lin-29, mab-10* and *ddx-23* mutant adults. **b,** Venn diagram depicting the overlap in genes downregulated in *lin-29, mab-10* and *ddx-23* mutant adults. **c.** Gene set enrichment analysis (using DAVID functional annotation tools) on the overlapping set of genes that are upregulated in in *lin-29, mab-10* and *ddx-23* mutant adults. **d,** Normalized gene expression abundance, calculated as $\log_2$(fragments per kilobase million (fpkm) +1), of multiple collagen genes that are up-regulated in *lin-29*, *mab-10*, and *ddx-23* mutant adult animals, as identified by RNA-Seq analysis. In wild-type animals, these collagen genes are expressed highly in L4 stage animals and decrease in expression in adults. Genotypes are depicted with the following colors: wild-type (black), *mab-10(tm2497)* (blue), *lin-29(n836)* (red), *ddx-23(n5705)* (purple). Error bars, mean value +/- SEM. $p < 0.01$ for all comparisons between wild-type vs. mutant. **e,** Normalized gene expression abundance, calculated as $\log_2$(fragments per kilobase million (fpkm) +1), of several *hedgehog*-related (*grd)* genes and their putative receptor (*ptr*) genes that are up-regulated in *lin-29*, *mab-10*, and *ddx-23* mutant adults, as identified by RNA-Seq analysis. Error bars, mean value +/- SEM. $p < 0.01$ for all comparisons between wild-type vs. mutant.

Source data for panels **d** and **e** are provided as a Source Data file.

Supplementary Table 1. *C. elegans* strains used in this study.

Strain	Genotype	Description	Related Figure(s)
N2	Wild-type (Bristol strain)	Reference wild-type strain	
MT22656	*maIs105* (Hawaiian strain)	Polymorphic Hawaiian strain with *maIs105[col-19::gfp]* used for genetic mapping and SNP analysis	
MT23456	*mab-10(n5909[mab-10::gfp]) II*	*mab-10* locus endogenously tagged with GFP	Fig. 1b, 1d, 1e, 1f, 3j, 3k, 3l
MT25264	*nEx2768*	Carries an extrachromosomal array of *Pceh-16::DDX-23(WT)cDNA-spGFPN::unc-54 3'UTR + Pceh-16::MAB-10cDNA-spGFPC::unc-54 3'UTR + Prab-3::mCherry*	Fig. 3d
MT25265	*nEx2769*	Carries an extrachromosomal array of *Pceh-16::DDX-23(WT)cDNA-spGFPN::unc-54 3'UTR + Pceh-16::empty-spGFPC::unc-54 3'UTR + Prab-3::mCherry*	Fig. 3d
MT25858	*nEx2931*	Carries an extrachromosomal array of *mab-10::mCherry + Pceh-16::mab-10::spGFPC + Pceh-16::ddx-23::spGFPN + Pmyo-2::mCherry*	Fig. 3e
MT25861	*nEx2934*	Carries an extrachromosomal array of *mab-10::mCherry + Pceh-16::empty::spGFPC + Pceh-16::ddx-23::spGFPN + Pmyo-2::mCherry*	Fig. 3e
MT26288	*n5909[mab-10::gfp]; ddx-23(n5703)*	*ddx-23(n5703)* mutant animal that carries the MAB-10 reporter (*n5909[mab-10::gfp]*)	Fig. 3j, 3k
MT25730	*n5909[mab-10::gfp]; ddx-23(n5705)*	*ddx-23(n5705)* mutant animal that carries the MAB-10 reporter (*n5909[mab-10::gfp]*)	Fig. 3j, 3k, 3l
MT26024	*ddx-23(n5705); maIs105; nEx2971*	*ddx-23(n5705)* mutant animal that carries the col-19::gfp reporter (*maIs105*) and an extrachromosomal array of *ddx-23(operon)::C. elegans-optimized human DDX23::ddx-23 3'UTR + myo-2p::mCherry*	Fig. 2f, Supp. Fig. 1f
MT26025	*ddx-23(n5705); maIs105; nEx2972*	*ddx-23(n5705)* mutant animal that carries the col-19::gfp reporter (*maIs105*) and an extrachromosomal array of *ddx-23(operon)::C. elegans-optimized human DDX23::ddx-23 3'UTR + myo-2p::mCherry*	Fig. 2f, Supp. Fig. 1f

Strain	Genotype	Description	Related Figure(s)
MT26011	*ddx-23(n5705); maIs105 ; nEx2958*	*ddx-23(n5705)* mutant animal that carries the col-19::gfp reporter (*maIs105*) and an extrachromosomal array of *ddx-23(operon)::DDX-23::tagRFP-T::ddx-23 3'UTR + myo-2p::mCherry*	Fig. 2c, Supp. Fig. 1d
MT25530	*mab-10(tm2497); ddx-23(n5705); maIs105; nEx2848*	*mab-10(tm2497); ddx-23(n5705)* mutant animal that carries the col-19::gfp reporter (*maIs105*) and an extrachromosomal array of *ddx-23(operon)::ddx-23::ddx-23 3'UTR + ttx-3promB::mCherry + rab-3p::mCherry::unc-54 3'UTR*	Fig. 2b, Supp. Fig. 1c
VT1367	*maIs105*	Carries the col-19::gfp reporter (*maIs105*)	Fig. 2b, 2c, 2g, 4c, Supp. Fig. 1c, Supp. Fig. 1d, Supp. Fig. 2a-d, Supp. Fig. 2f, Supp. Fig. 4
MT23825	*ddx-23(n5705); maIs105*	*ddx-23(n5705)* mutant animal that carries the col-19::gfp reporter (*maIs105*)	Fig. 2c, 2g, 4c, Supp. Fig. 1d, Supp. Fig. 2f, Supp. Fig. 4
MT23818	*ddx-23(n5703); maIs105*	*ddx-23(n5703)* mutant animal that carries the col-19::gfp reporter (*maIs105*)	Fig. 2c, 2g, Supp. Fig. 1d
MT19733	*mab-10(tm2497); maIs105*	*mab-10(tm2497)* mutant animal that carries the col-19::gfp reporter (*maIs105*)	Fig. 2b, 2g, 4c, Supp. Fig. 1c, Supp. Fig. 4
MT23824	*mab-10(tm2497); ddx-23(n5705); maIs105*	*mab-10(tm2497); ddx-23(n5705)* mutant animal that carries the col-19::gfp reporter (*maIs105*)	Fig. 2b, 2g, Supp. Fig. 1c
MT23817	*mab-10(tm2497); ddx-23(n5703); maIs105*	*mab-10(tm2497); ddx-23(n5703)* mutant animal that carries the col-19::gfp reporter (*maIs105*)	Fig. 2b, Supp. Fig. 1c
MT24358	*lin-29(n836); maIs105*	*lin-29(n836)* mutant animal that carries the col-19::gfp reporter (*maIs105*)	Fig. 2g, 4c, Supp. Fig. 4
MT24153	*n6026; maIs105*	*ddx-23(n6026)[I383F]* mutant animal that carries the col-19::gfp reporter (*maIs105*)	Supp. Fig. 2a-d
MT24154	*n6027; maIs105*	*ddx-23(n6027)[S365N]* mutant animal that carries the col-19::gfp reporter (*maIs105*)	Supp. Fig. 2a-d
MT22079	*wIs78*	Carries the *wIs78[ajm-1::gfp + scm::gfp]* reporter	Supp. Fig. 2g

Strain	Genotype	Description	Related Figure(s)
MT19521	*mab-10(tm2497); wIs78; him-5(e1467ts)*	*mab-10(tm2497)* mutant animal that carries the *wIs78[ajm-1::gfp + scm::gfp]* reporter	Supp. Fig. 2g
MT24251	*ddx-23(n5705); wIs78*	*ddx-23(n5705)* mutant animal that carries the *wIs78[ajm-1::gfp + scm::gfp]* reporter	Supp. Fig. 2g
MT24252	*mab-10(tm2497); ddx-23(n5705); wIs78*	*mab-10(tm2497); ddx-23(n5705)* mutant animal that carries the *wIs78[ajm-1::gfp + scm::gfp]* reporter	Supp. Fig. 2g
MT24355	*ddx-23(n6092[tagRFP-T LoxP 3xFlag::ddx-23])*	*ddx-23* locus endogenously tagged with tagRFP-T	Supp. Fig. 2e
MT24723	*n5909[mab-10::gfp]; ddx-23(n6092 [tagRFP-T LoxP 3xFlag::ddx-23])*	Strain carrying the MAB-10 reporter (*n5909[mab-10::gfp]*) and the DDX-23 reporter (*n6092 [tagRFP-T LoxP 3xFlag::ddx-23]*)	Fig. 3a
MT23457	*lin-29(n5908[lin-29::gfp])*	*lin-29* locus endogenously tagged with GFP	Fig. 4b
MT26078	*lin-29(n5908[lin-29::gfp]); nEx3004*	Strain carrying the LIN-29 reporter (*n5908[lin-29::gfp]*) and an extrachromosomal array of *mab-10p::MAB-10::mCherry + myo-2p::mCherry*	Fig. 4a
MT23550	*mab-10(tm2497) n5908[lin-29::gfp]*	*mab-10(tm2497)* mutant animal carrying the LIN-29 reporter (*n5908[lin-29::gfp]*)	Fig. 4b
MT24317	*n5908[lin-29::gfp]; ddx-23(n5705)*	*ddx-23(n5705)* mutant animal carrying the LIN-29 reporter (*n5908[lin-29::gfp]*)	Fig. 4b
MT24362	*mab-10(tm2497) n5908[lin-29::gfp]; ddx-23(n5705)*	*mab-10(tm2497); ddx-23(n5705)* mutant animal carrying the LIN-29 reporter (*n5908[lin-29::gfp]*)	Fig. 4b

References

1. Peng, K., Radivojac, P., Vucetic, S., Dunker, A. K. & Obradovic, Z. Length-dependent prediction of protein intrinsic disorder. *BMC Bioinformatics* **7**, 208 (2006).

2. Kuznetsova, I. M., Turoverov, K. K. & Uversky, V. N. What macromolecular crowding can do to a protein. *Int. J. Mol. Sci.* **15**, 23090–23140 (2014).
