# Supplementary material for: A DEAD-box helicase drives the partitioning of a pro-differentiation NAB protein into nuclear foci
Source: Nat Commun. 2023 Oct 18;14:6593. doi: 10.1038/s41467-023-42345-9 (PMC10584935; doi:10.1038/s41467-023-42345-9)
Supplement: Supplementary file 2 — Reporting Summary [file 41467_2023_42345_MOESM2_ESM.pdf]

nature portfolio

Corresponding author(s): H. Robert Horvitz
Last updated by author(s): 9/24/2023

Reporting Summary

Nature Portfolio wishes to improve the reproducibility of the work that we publish. This form provides structure for consistency and transparency in reporting. For further information on Nature Portfolio policies, see our Editorial Policies and the Editorial Policy Checklist.

Statistics

For all statistical analyses, confirm that the following items are present in the figure legend, table legend, main text, or Methods section.

n/a	Confirmed	
☐	☒	The exact sample size (*n*) for each experimental group/condition, given as a discrete number and unit of measurement
☐	☒	A statement on whether measurements were taken from distinct samples or whether the same sample was measured repeatedly
☐	☒	The statistical test(s) used AND whether they are one- or two-sided<br>*Only common tests should be described solely by name; describe more complex techniques in the Methods section.*
☒	☐	A description of all covariates tested
☐	☒	A description of any assumptions or corrections, such as tests of normality and adjustment for multiple comparisons
☐	☒	A full description of the statistical parameters including central tendency (e.g. means) or other basic estimates (e.g. regression coefficient) AND variation (e.g. standard deviation) or associated estimates of uncertainty (e.g. confidence intervals)
☐	☒	For null hypothesis testing, the test statistic (e.g. *F*, *t*, *r*) with confidence intervals, effect sizes, degrees of freedom and *P* value noted<br>*Give P values as exact values whenever suitable.*
☒	☐	For Bayesian analysis, information on the choice of priors and Markov chain Monte Carlo settings
☒	☐	For hierarchical and complex designs, identification of the appropriate level for tests and full reporting of outcomes
☒	☐	Estimates of effect sizes (e.g. Cohen's *d*, Pearson's *r*), indicating how they were calculated

Our web collection on statistics for biologists contains articles on many of the points above.

Software and code

Policy information about availability of computer code

Data collection	RNA-seq libraries were prepared for sequencing using standard Illumina protocols (using the NeoPrep system) and run on Illumina NextSeq 500.<br>Nomarski DIC and epifluorescence micrographs were obtained using a Zeiss Axio Imager Z2 compound microscope and Zen Blue (Zeiss) software. Confocal microscopy was performed using the Zeiss LSM 800 instrument and images were obtained using the Zen Blue 2.0 (Zeiss) software.
Data analysis	For RNA-seq data analysis, quality control was carried out by MIT BMC/BCC in-house pipeline including sequencing error rate estimation, sequencing reads complexity estimation, fastqc report, and sample contamination estimation. RNA-Seq mapping was performed against ws258/WBcel235 reference for Caenorhabditis elegans sequences using STAR/2.5.3a. Gene expression was quantified using RSEM v. 1.3.0. Differential gene expression analysis was performed using DESeq2.<br>ImageJ 1.52i was used to determine fluorescence signal intensity of C. elegans reporters from micrographs. Adobe Illustrator CS4 software was used for preparing images for final publication. GraphPad Prism 6 software was used to perform all statistical analysis and graph generation.

For manuscripts utilizing custom algorithms or software that are central to the research but not yet described in published literature, software must be made available to editors and reviewers. We strongly encourage code deposition in a community repository (e.g. GitHub). See the Nature Portfolio guidelines for submitting code & software for further information.

Data

Policy information about availability of data

All manuscripts must include a data availability statement. This statement should provide the following information, where applicable:

- Accession codes, unique identifiers, or web links for publicly available datasets
- A description of any restrictions on data availability
- For clinical datasets or third party data, please ensure that the statement adheres to our policy

All data supporting the findings of this paper are provided in the main text, supplementary materials, and source data file. The RNAseq dataset generated in this study has been deposited in the NCBI GEO database under accession code GSE151399. Caenorhabditis elegans ws258 / WBcel235 reference sequences were used in this study. Further information and requests for resources and reagents are available upon request to the corresponding author.

Research involving human participants, their data, or biological material

Policy information about studies with human participants or human data. See also policy information about sex, gender (identity/presentation), and sexual orientation and race, ethnicity and racism.

Reporting on sex and gender	N/A
Reporting on race, ethnicity, or other socially relevant groupings	N/A
Population characteristics	N/A
Recruitment	N/A
Ethics oversight	N/A

Note that full information on the approval of the study protocol must also be provided in the manuscript.

Field-specific reporting

Please select the one below that is the best fit for your research. If you are not sure, read the appropriate sections before making your selection.

[x] Life sciences [ ] Behavioural & social sciences [ ] Ecological, evolutionary & environmental sciences

For a reference copy of the document with all sections, see nature.com/documents/nr-reporting-summary-flat.pdf

Life sciences study design

All studies must disclose on these points even when the disclosure is negative.

Sample size	No statistical methods were used to predetermine sample size. For analysis of reporter intensities and seam cell counts in C. elegans, we found that 5 randomly selected animals largely reflected the variance seen within each genotype of animals. We therefore used at least 5 animals per treatment/mutant group for all such analyses. For in vitro protein interaction assays, we examined 3 independent experiments and the results from all 3 experiments led to the same conclusion.
Data exclusions	No data were excluded from analysis.
Replication	All experiments using C. elegans animals were performed at least three independent times and successfully replicated each time. Similarly, in vitro findings using recombinant proteins were successfully replicated at least three times.
Randomization	C. elegans animals were randomly chosen for imaging and quantification for each condition in each experiment.
Blinding	We did not perform blinding experiments because mutant animals display abnormal phenotypes that are obvious and hence blinding would not work.

Reporting for specific materials, systems and methods

We require information from authors about some types of materials, experimental systems and methods used in many studies. Here, indicate whether each material, system or method listed is relevant to your study. If you are not sure if a list item applies to your research, read the appropriate section before selecting a response.

Materials & experimental systems

n/a	Involved in the study	
☒	☐	Antibodies
☒	☐	Eukaryotic cell lines
☒	☐	Palaeontology and archaeology
☐	☒	Animals and other organisms
☒	☐	Clinical data
☒	☐	Dual use research of concern
☒	☐	Plants

Methods

n/a	Involved in the study	
☒	☐	ChIP-seq
☒	☐	Flow cytometry
☒	☐	MRI-based neuroimaging

Animals and other research organisms

Policy information about studies involving animals; ARRIVE guidelines recommended for reporting animal research, and Sex and Gender in Research

Laboratory animals	Caenorhabditis elegans. Strain names: N2, MT22656, MT23456, MT25264, MT25265, MT25858, MT25861, MT26288, MT25730, MT26024, MT26025, MT26011, MT25530, VT1367, MT23825, MT23818, MT19733, MT23824, MT23817, MT24358, MT24153, MT24154, MT22079, MT19521, MT24251, MT24252, MT24355, MT24723, MT26078, MT23457, MT23550, MT24317, MT24362. All strains were tested at L4 or one-day old adults. More details on the strains used in this study are included in Supplementary Table 1.
Wild animals	Study did not involve animals caught in the wild.
Reporting on sex	All animals used in this paper (unless otherwise stated) were hermaphrodite as C. elegans primarily reproduce as hermaphrodites.
Field-collected samples	Study did not involve samples collected from the field.
Ethics oversight	Massachusetts Institute of Technology

Note that full information on the approval of the study protocol must also be provided in the manuscript.
